# Gut microbiota alters host bile acid metabolism to contribute to intrahepatic cholestasis of pregnancy

**DOI:** 10.1038/s41467-023-36981-4

**Published:** 2023-03-09

**Authors:** Bo Tang, Li Tang, Shengpeng Li, Shuang Liu, Jialin He, Pan Li, Sumin Wang, Min Yang, Longhui Zhang, Yuanyuan Lei, Dianji Tu, Xuefeng Tang, Hua Hu, Qin Ouyang, Xia Chen, Shiming Yang

**Affiliations:** 1 Department of Gastroenterology, Xinqiao Hospital, Army Medical University, Chongqing, China; 2 Department of Obstetrics and Gynecology, First People’s Hospital of Foshan, Foshan, Guangdong China; 3 Department of Obstetrics and Gynecology, Xinqiao Hospital, Army Medical University, Chongqing, China; 4 Laboratory Medicine Center, Xinqiao Hospital, Army Medical University, Chongqing, China; 5 Department of Pathology, Xinqiao Hospital, Army Medical University, Chongqing, China; 6 College of Pharmacy, Army Medical University, Chongqing, China; 7 Chongqing Institute for Brain and Intelligence, Guangyang Bay Laboratory, Chongqing, China; 8 Chongqing Municipality Clinical Research Center for Gastroenterology, Chongqing, China

**Keywords:** Microbiome, Liver diseases

## Abstract

Intrahepatic cholestasis of pregnancy (ICP) is a female pregnancy-specific disorder that is characterized by increased serum bile acid and adverse fetal outcomes. The aetiology and mechanism of ICP are poorly understood; thus, existing therapies have been largely empiric. Here we show that the gut microbiome differed significantly between individuals with ICP and healthy pregnant women, and that colonization with gut microbiome from ICP patients was sufficient to induce cholestasis in mice. The gut microbiomes of ICP patients were primarily characterized by *Bacteroides fragilis* (*B. fragilis*), and *B. fragilis* was able to promote ICP by inhibiting FXR signaling via its BSH activity to modulate bile acid metabolism. *B. fragilis*-mediated FXR signaling inhibition was responsible for excessive bile acid synthesis and interrupted hepatic bile excretion to ultimately promote the initiation of ICP. We propose that modulation of the gut microbiota-bile acid-FXR axis may be of value for ICP treatment.

## Abstract (web summary)

Intrahepatic cholestasis of pregnancy (ICP) is a liver disease that sometimes develops during pregnancy and is characterized by increased serum bile acid levels. Here the authors report that the gut microbiome species B. fragilis is enriched in patients with ICP and promotes ICP development in mice via inhibition of signalling though the bile acid receptor FXR.

## Introduction

Intrahepatic cholestasis of pregnancy (ICP) is the most common pregnancy-related liver disease, which predominantly occurs in the second or third trimester and is mainly characterized by maternal pruritus and increased levels of serum bile acid and liver transaminases^1^. ICP is mainly associated with increased adverse perinatal outcomes, such as spontaneous preterm delivery; fetal distress, intrauterine death and growth restriction; low Apgar score and meconium-stained fluid with a small degree of maternal risk^2,3^. The aetiology and pathophysiology of ICP remain poorly understood, and therapies have been largely empiric. Ursodeoxycholic acid (UDCA) is commonly used to treat ICP and is effective in ameliorating pruritus by reducing alanine aminotransferase in ICP^4–6^. However, previous studies have shown that there is insufficient evidence to recommend UDCA to improve fetal outcomes and bile acid levels^6,7^, leading to controversy about its use in ICP.

The gut microbiota in the gastrointestinal tract are able to impact the functions related to immune, metabolic and inflammatory diseases^8,9^ and their composition can be shaped by various environmental factors, such as diet, immune system, host genetics, and hormones^10–14^. It is well known that the human body undergoes substantial immunological, hormonal and metabolic changes during a normal pregnancy^15,16^. Previous studies have shown that the gut microbiota in healthy pregnant women undergoes profound alterations from the first to the third trimester^17–19^. Moreover, gut microbiota dysbiosis may be sufficient to induce disease even in otherwise nonpredisposed individuals^20^. Our group recently reported that the gut microbiota of individuals with preeclampsia (PE) were dysbiotic and that microbiota transplantation from patients with PE could promote a PE-like phenotype in pregnant mice^21^. Additionally, previous studies have revealed a close link between microbial dysbiosis and many pregnancy-related diseases^22–24^. This highlights the importance of understanding microbiome changes during pregnancy, which might offer a promising approach of preemptively modulating the microbiota before conception or during early pregnancy to reduce the risk of adverse outcomes. A previous study revealed that there was a notable metabolic change during the gestation period that induced elevated serum bile acids in pregnancy^25^. Primary bile acids synthesized from cholesterol in the liver are conjugated with glycine and taurine and secreted into the intestine. Then, the primary bile acids are transformed into secondary bile acids, and the deconjugation of the bile acids is mediated by intestinal bacteria^26^, with FXR being the key receptor that regulates hepatic bile acid biosynthesis, transport and secretion^26^.

Recent studies using 16S rRNA sequencing have revealed that compared to healthy controls, patients with ICP have different gut microbiota profiles^27,28^. However, there is insufficient evidence to resolve the cause-and-effect relationships and to determine whether gut microbiota changes are a consequence of disease or contribute to the development of ICP. In particular, the particular species of gut microbiota involved and the underlying mechanism by which the gut microbiota impact ICP pathogenesis are still unknown. Herein, we aimed to determine whether ICP is associated with a specific gut microbiome profile and to clarify the role of the gut microbiota in ICP pathogenesis.

Our present study showed that a distinct microbial profile was present in ICP patients, and that the abundance of *Bacteroides fragilis* was substantially increased in individuals with ICP. Transplantation of the faecal microbiota from patients with ICP was sufficient to promote an ICP-like phenotype. We further suggest a mechanism involving microbe-mediated bile acid metabolism and discuss how it might regulate FXR signaling and the incidence of ICP. Our data highlight the importance of the gut microbiota in ICP development and provide a potential target for the clinical management of ICP.

## Results

### Gut microbiota from patients with ICP is altered significantly and sufficiently to promote intrahepatic cholestasis of pregnancy in mice

**Fig. 1:**
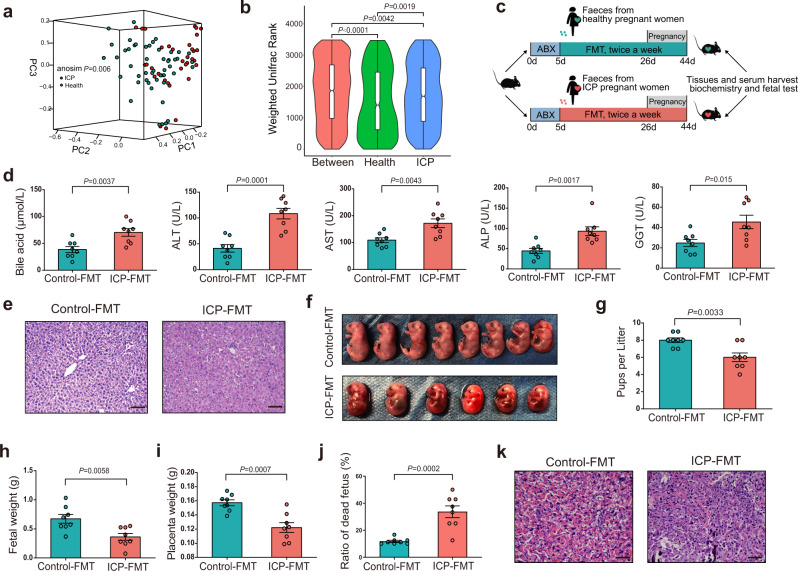
Gut microbiota from ICP patients is altered significantly and sufficient to promote ICP in mice. **a** Weighted Unifrac PCoA (principal coordinate analysis) plot of individuals with ICP patients and healthy controls. The ANOSIM test was used to calculate the significance of dissimilarity (ANOSIM, *P* = 0.001). **b** A truncated violin plot shows the comparison of Weighted Unifrac range of samples between ICP patients and healthy controls. The notch in the middle of the box represents the median. The top and bottom of box represent the 75th and 25th quartiles, respectively. The upper and lower whiskers extended 1.5× the interquartile range from the upper edge and lower edge of the box represent maximum and minimum, respectively. With a truncated violin plot, the curve of the violin extends only to the minimum and maximum values in the data set. Outlier values were not shown in this plot. *n* = 50 individuals with ICP, *n* = 41 individuals in healthy controls. The ANOSIM test was used to calculate the significance of dissimilarity. The exact *P* values were shown. **c** Experimental schematic: following antibiotics treatment, the recipient mice were transplanted with fecal samples from ICP patients or healthy controls. Tissues and samples were collected at E18d. **d** Analysis of serum levels of total bile acids, ALT, AST, ALP, and GGT (*n* = 8 mice per group). Data are presented as mean ± SEM. *P* values were determined by two-tailed Student’s *t*-test. **e** Hematoxylin and eosin staining of representative livers. Scale bar: 100 μm. **f**, **g** Number of pups per litter (*n* = 8 mice per group). The representative fetuses are from the same litter with the same gestational age. **h**–**j** Fetal weight (**h**), placenta weight (**i**) and ratio of dead fetus (**j**) in the two groups (*n* = 8 mice per group). *P* values were determined by two-tailed Student’s *t*-test for **g**–**j**. **k** Placenta tissues were stained with H&E. Representative images were shown. Scale bar: 50 μm. Data are presented as mean ± SEM for **g**–**j**. FMT fecal microbiota transplantation, ABX antibiotics cocktail, PC principal coordinate analysis, ANOSIM analysis of similarities. Source data are provided as a Source Data file.

BMI- and age-matched pregnant women with ICP (*n* = 50) and control pregnant women (*n* = 41) were recruited. We then carried out whole-genome shotgun sequencing on faecal samples from these healthy pregnant women and patients with ICP (for demographic and clinical characteristics see Supplementary Table 1). A metagenomic dataset with an average of 40,420,272 ± 3,544,978 paired-end reads per sample was obtained. Raw reads were preprocessed using KneadData to eliminate human DNA sequences and to filter sequences with poor quality, which removed 5.26% of the reads. Ultimately, 40,420,272 ± 3,544,978 reads per sample were obtained. The sequences were analysed with MetaPhLan3 implemented within the HUMAnN3 pipeline. We selected a total of 904 clades including 12 phyla (L2), 23 classes (L3), 37 orders (L4), 73 families (L5), 192 genera (L6) and 567 species (L7). Alpha diversity was calculated to evaluate the differences between healthy controls and ICP patients. No significant difference in alpha diversity was observed among the taxon levels (Wilcoxon rank-sum test; Supplementary Fig. 1). For beta diversity, weighted Unifrac distances were analysed and plotted by principal coordinate analysis (PCoA) (ANOSIM; *P* = 0.006; Fig. 1a). The weighted Unifrac rank between the two groups was much higher than that within each group (Fig. 1b).

To investigate the role of the gut microbiota in ICP, stool samples from healthy controls or patients with ICP was transplanted into recipient mice (Fig. 1c). We further collected faeces from mice receiving ICP stool transplants and from mice receiving healthy donor stool transplants and performed 16S rRNA sequencing to validate the fidelity of the microbiota transplantation. The gut microbiota of mice receiving ICP stool transplants was altered significantly compared to that of control mice (Supplementary Fig. 2). Compared with mice receiving stool transplants from healthy pregnant women, mice receiving ICP stool transplants displayed increased levels of total bile acid (TBA), aspartate transaminase (AST), alanine aminotransferase (ALT), alkaline phosphatase (ALP) and gamma-glutamyl transpeptidase (GGT) (Fig. 1d). The hepatic tissue in mice receiving stool transplants from healthy controls exhibited a normal morphology, whereas cytoplasm rarefaction, nuclear condensation, vacuolar degeneration, portal oedema and a loss of hepatic structure in periportal areas were observed in hepatic tissues of mice receiving stool transplants with microbiota from patients with ICP (Fig. 1e). The number of live pups (Fig. 1f, g), fetal weight (Fig. 1h) and placental weight (Fig. 1i) were significantly decreased in the mice receiving stool transplants from women with ICP, and the dead fetus rate (Fig. 1j) in these mice was higher than the rate in mice receiving transplants of stool from healthy controls. Histomorphological analysis revealed that fecal transplantation from individuals with ICP resulted in intracellular oedema and severe atrophy of trophoblasts in the placenta (Fig. 1k). Thus, our data demonstrated that microbiota transplantation from patients with ICP into mice could transfer ICP-related phenotypes.

### *B. fragilis* induced intrahepatic cholestasis in pregnant mice

**Fig. 2:**
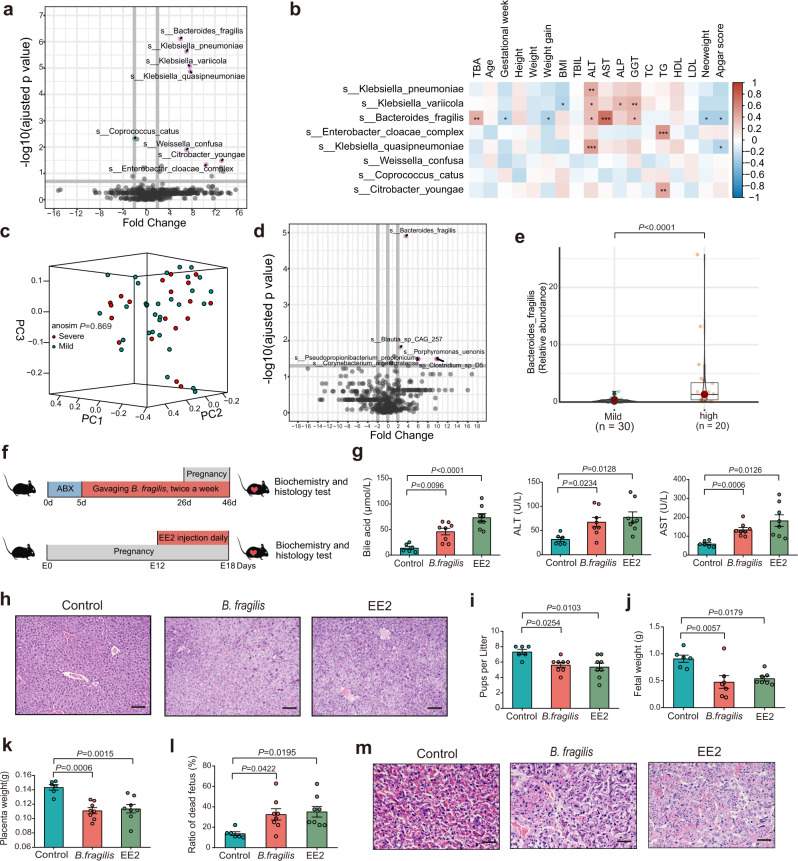
*B. fragilis* induced intrahepatic cholestasis of pregnancy in mice. **a** Differentially enriched bacteria between ICP patients and healthy controls. Pink dots represent bacteria with higher abundance in samples of ICP patients. Blue dots represent bacteria with higher abundance in samples of healthy controls. Grey dots represent bacteria with no significant difference between the two groups. *P* values were adjusted by Benjamini & Hochberg (BH) method to control FDR. FDR-adjusted *P* < 0.05 was shown. **b** Correlation heatmap of differentially enriched bacteria with the clinical characteristics. Pearson correlation analysis with FDR-adjusted *P* value. *P < 0.05; **P < 0.01; ***P < 0.001. **c** Weighted Unifrac PCoA plot of samples between the severe and mild groups in ICP patients. The ANOSIM test was used to calculate the significance of dissimilarity (ANOSIM, *P* = 0.869). **d** Differentially enriched bacteria between the severe and mild groups in ICP patients. Pink dots represent bacteria with relatively higher abundance in severe ICP patients. Grey dots represent bacteria with no significant difference between the two groups. *P* values were adjusted by Benjamini & Hochberg (BH) method to control FDR. FDR-adjusted *P* < 0.05 was shown. **e** The comparison of *B. fragilis* abundance between the two groups. The horizontal bar within box represents median. The top and bottom of box represent 75th and 25th quartiles, respectively. The upper and lower whiskers extended 1.5× the interquartile range from the upper edge and lower edge of the box represent maximum and minimum, respectively. *P* value was determined by two-tailed Mann–Whitney test. **f** Experimental schematic for gavaging *B. fragilis*. **g** Analysis of serum levels of total bile acids, ALT and AST in each group. Data are presented as mean ± SEM. *P* values were determined by ordinary one-way ANOVA with Tukey’s correction or Welch ANOVA with Games-Howell’s multiple comparisons test. *n* = 6 mice in control group, *n* = 8 mice in *B. fragilis* and EE2 group. **h** Representative images of H&E staining of livers in eac**h** group. Scale bar: 100 μm. **i**–**l** Number of pups per litter (**i**) in each group. *n* = 6 in control group, *n* = 8 in *B. frag****i****lis* and EE2 group. Number of fetal weight (**j**) in each group. *n* = 6 in control group, *n* = 7 in *B. fragilis* and EE2 group. Number of placenta weight (**k**) and ratio of dead fetus (**l**) in each group. *n* = 6 in control group, n = 8 in *B. fragi****l****is* and EE2 group. Data are presented as mean ± SEM for **i**–**l**. *P* values were determined by ordinary one-way ANOVA with Tukey’s correction for **i**–**l**. **m** Representative images of H&E staining of placenta in each group. Scale bar: 50 μm. ANOSIM analysis of similarities, EE2 17α-ethynylestradiol; Source data are provided as a Source Data file.

We further sought to identify the differentiating patterns in bacterial taxa between the two groups and to explore the microbial species associated with ICP parameters. The microbiomes of individuals with ICP were primarily characterized by *Bacteriodes fragilis*, *Klebsiella pneumoniae*, *Klebsiella variicola, Klebsiella quasipneumoniae, Weissella confusa, Citrobacter youngae,* and *Enterobacter cloacae* (Wilcoxon rank-sum test; Fig. 2a). In addition, some of these microbial species were associated with ICP clinical parameters, such as levels of TBA, ALT, AST, ALP, GGT, birth weight and Apgar score (Fig. 2b). To further evaluate the relationship between microbiota and ICP severity, we divided the patients with ICP into two groups on the basis of TBA levels (mild group, serum TBA range 10–39.9 μmol/L; severe group serum TBA ≥ 40 μmol/L). We compared the demographic characteristics between the mild and severe group and found that there were significant differences in TBA levels, ALT, ALP, GGT, neonate weight and Apgar score (Supplementary Table 2). Although there was no notable difference in the alpha and beta diversity between the mild and severe groups (Supplementary Fig. 3; Fig. 2c), we observed that the microbiomes of individuals with severe ICP were primarily characterized by *Bacteriodes fragilis* (*B. fragilis*) (Fig. 2d), and that the abundance of *B. fragilis* was markedly increased in the severe group compared to the mild group (Fig. 2e). *B. fragilis* was positively correlated with the levels of TBA, ALT and AST but negatively related to gestational age, birth weight and Apgar score (Fig. 2b).

We further tested whether *B. fragilis* might play an important role in the development of ICP. Hence, we transplanted *B. fragilis* into C57BL/6 mice using oral gavage after antibiotic-based microbiota depletion (Fig. 2f). The EE2-induced ICP mouse model was used as the positive control (Fig. 2f). Notably, we observed that colonization of *B. fragilis* caused increased levels of TBA (Fig. 2g) and cholestatic liver injury (Fig. 2h; Supplementary Fig. 4a–c), disrupted normal fetal growth (Fig. 2i–l; Supplementary Fig. 4d), and induced histopathological abnormalities of the placenta in recipient mice (Fig. 2m). Our data suggest that *B. fragilis* could promote intrahepatic cholestasis in a pregnant mouse model.

### *B. fragilis* promotes ICP through its BSH activity mediating bile acid metabolism

**Fig. 3:**
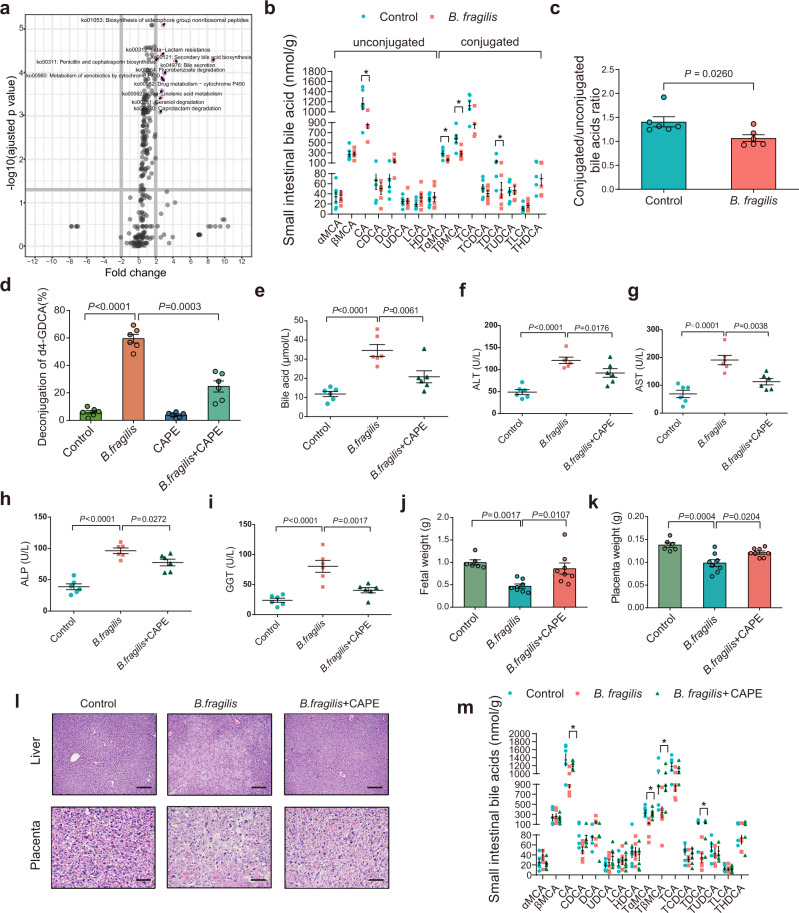
*B. fragilis* promotes the ICP through its BSH activity mediating bile acid metabolism. **a** Kyoto Encyclopedia of Genes and Genomes (KEGG) annotation of key altered metabolic pathways in individuals with ICP. *P* values were adjusted by Benjamini & Hochberg (BH) method to control FDR. FDR-adjusted *P* < 0.05 was shown. **b** Levels of small intestinal bile acids in *B. fragilis* transplanted mice or control group (*n* = 6 mice per group). *P* values were determined by two-tailed Student’s *t*-test. *P < 0.05. **c** Conjugated and unconjugated bile acids in *B. fragilis* transplanted mice or control group. *n* = 6 per group. *P* values were determined by two-sided Mann–Whitney test. **d** Hydrolysis efficiency of GDCA by *B. fragilis* with or without CAPE in vitro. *P* values were determined by Welch ANOVA with Games-Howell’s multiple comparisons test. *n* = 6 per group. **e**–**i** Mice were divided into three groups (control, *B. fragilis* and *B. fragilis* + CAPE). Analysis of serum levels of total bile acids (**e**), ALT (**f**), AST (**g**), ALP (**h**) and GGT (**i**) in eac**h** group. *n* = 6 per group. *P* values were determined by ordinary one-way ANOVA with Tukey’s correction. Fetal weight (**j**) and placenta weight (**k**) in each group (*n* = 6 in control group; *n* = 8 in *B. fragilis* group and *B. fragilis* + CAPE group). *P* values were determined by ordinary one-way ANOVA with Tukey’s correction. **l** Representative images of H&E staining of livers and placenta in each group. Scale bar = 100 μm for liver; scale bar = 50 μm for placenta. **m** Levels of small intestinal bile acids in indicated groups (*n* = 6 per group). *P* value was determined by one-way ANOVA with Tukey’s correction. *P < 0.05. Data are presented as mean ± SEM for **b**–**k**, **m**. CAPE caffeic acid phenethyl ester. Source data are provided as a Source Data file.

Since there was a difference in microbiome composition between healthy controls and ICP patients, we further investigated the functional differences by analysing the functional pathways. We performed Kyoto Encyclopedia of Genes and Genomes (KEGG) pathway analysis and observed that several pathways were enriched in the ICP patients. Among these pathways, bile acid metabolism was the top metabolic pathway affected by the ICP microbiome (Fig. 3a). We found that the serum concentrations of most conjugated bile acids were much higher in patients with ICP (Supplementary Fig. 5a), and that several conjugated bile acids were decreased in fecal samples from patients with ICP (Supplementary Fig. 5b, c). To identify whether fecal transplantation from ICP patients or *B. fragilis* colonization was responsible for the bile acid metabolism patterns, we performed targeted metabolomics analyses. We observed that fecal transplantation from patients with ICP could decrease the ratio of conjugated bile acids (Supplementary Fig. 6). We also found that the levels of conjugated bile acids in the small intestine were dramatically decreased in *B. fragilis*-colonized mice (Fig. 3b, c), with a significant reduction in tauro-α-muricholic acid (TαMCA), tauro-β-muricholic acid (TβMCA), and taurodeoxycholic acid (TDCA).

It is well known that BSH enzymes in microbial genera can deconjugate glycine or taurine-conjugated bile acids^26^. Furthermore, we investigated whether *B. fragilis* with high BSH activity could directly mediate the deconjugation of bile acids in vitro. We observed that *B. fragilis* could mediate the deconjugation of conjugated bile acids which was attenuated by the BSH inhibitor caffeic acid phenethyl ester (CAPE) (Fig. 3d). *B. fragilis* had lower activity in conjugated CA or CDCA compared to conjugated DCA (Supplementary Fig. 7), demonstrating the substrate specificity of the *B. fragilis* BSH enzyme. To explore the function of BSH activity enriched in *B. fragilis* in the development of the ICP phenotype, CAPE was added during the *B. fragilis* gavage (Supplementary Fig. 8a). CAPE significantly reversed *B. fragilis*-induced cholestatic liver injury (Fig. 3e–i; Supplementary Fig. 8b), fetal growth inhibition (Fig. 3j, k; Supplementary Fig. 8c), and placental abnormalities (Fig. 3l). Similarly, CAPE supplementation attenuated *B. fragilis-*induced deconjugation of conjugated bile acids in the mouse small intestine in vivo (Fig. 3m). Thus, our results suggested that *B. fragilis* could promote the ICP phenotype through its deconjugation of BSH activity.

### *B. fragilis* suppressed FXR signaling by mediating bile acid metabolism to increase hepatic bile acid accumulation

**Fig. 4:**
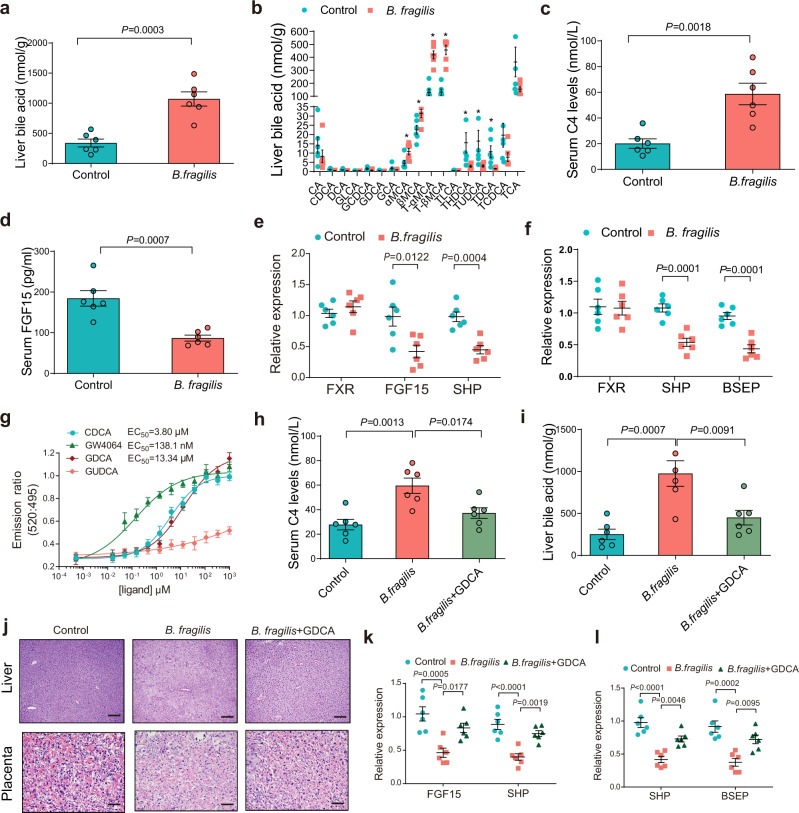
*B. fragilis* suppressed FXR signaling through mediating bile acid metabolism to increase hepatic bile acids accumulation. **a** Liver total bile acid levels in *B. fragilis* transplanted mice or control group (*n* = 6 per group). **b** Hepatic bile acids profiles in *B. fragilis* transplanted mice or control group (*n* = 6 per group). *P* values were determined by two-tailed Student’s *t*-test for **a**, **b**. *P < 0.05. **c**, **d** Serum C4 levels (**c**) and FGF15 levels (**d**) in *B. fragilis* transplanted mice or control group (*n* = 6 per group). *P* values were determined by two-tailed Student’s *t*-test. e, f Relative expression of intestinal (**e**) and hepatic (**f**) FXR mRNA and its target genes in mice colonized with *B. fragilis* or control (*n* = 6 per group). *P* values were determined by two-tailed Student’s *t*-test. **g** TR-FRET FXR coactivator recruitment assay to assess the action of GDCA on FXR; CDCA and GW4064 was used as positive control. GUDCA was used as negative control (*n* = 3 per group). **h** Serum C4 levels in each group (*n* = 6 per group). **i** Liver total bile acid levels in each group (*n* = 6 per group). *P* values were determined by one-way ANOVA with Tukey’s correction for **h**–**j** Representative images of H&E staining of livers and placenta in each group. Scale bar = 100 μm for liver; scale bar = 50 μm for placenta. **k**, **l** Relative expression of intestinal FXR target genes (**k**) and hepatic FXR target genes (**l**) in indicated groups. *n* = 6 per group. *P* values were determined by one-way ANOVA with Tukey’s correction. Data are presented as mean ± SEM for **a**–**i**, **k**, **l**. Source data are provided as a Source Data file.

Since massive bile acid accumulation is closely associated with the incidence and development of cholestatic liver diseases, we previously investigated and found that *B. fragilis* could increase the total serum bile acid level. We further observed that hepatic bile acid levels were significantly increased by *B. fragilis* colonization in mice (Fig. 4a). We found that hepatic levels of T-αMCA, T-βMCA and TCA were significantly elevated in *B. fragilis*-colonized mice (Fig. 4b). Notably, *B. fragilis* caused a significant increase in the serum 7α-hydroxy-4-cholesten-3-one (C4) level, which is a surrogate of bile acid synthesis (Fig. 4c). We also observed that the increased conjugated bile acids induced by *B. fragilis* colonization could activate sphingosine-1-phosphate receptor 2 (S1PR2) and promote inflammation in the liver (Supplementary Fig. 9). Since bile acids mainly exert their functions by interacting with receptors such as FXR, we further tested intestinal and liver FXR signaling. In the intestine, FXR activation induced FGF15 expression in mice, and FGF15 bound to the FGF receptor/β-Klotho complex on hepatocytes and repressed bile acid synthesis. Our results showed that FGF15 levels in the serum of mice receiving transplants with ICP microbiota were dramatically decreased (Supplementary Fig. 10a), and the expression levels of downstream genes of FXR were markedly suppressed in the ileum and liver of ICP microbiota-transplanted mice (Supplementary Fig. 10b, c), suggesting that intestinal and hepatic FXR signaling was inhibited by ICP gut microbiota transplantation. Additionally, the expression of *FGF15* and *Shp* in ileal tissues was significantly reduced by *B. fragilis* colonization (Fig. 4d, e). The expression of FXR target genes in hepatic tissues was similarly reduced by *B. fragilis* colonization (Fig. 4f). These data suggest that *B. fragilis* administration markedly suppressed intestinal and hepatic FXR signaling.

Since we previously found that *B. fragilis* could directly mediate the deconjugation of GDCA through its BSH activity, we further investigated whether the FXR signaling change resulted from *B. fragilis*-related bile acid metabolism. We found that GDCA could substantially induce FXR transactivation, which was inhibited by GUDCA (Supplementary Fig. 11a). Furthermore, we observed that GDCA induced the expression levels of the FXR target genes *FGF19* and *Shp* in a dose-dependent manner in the human intestinal Caco-2 cell line (Supplementary Fig. 11b). To further clarify the interaction between GDCA and FXR, in silico molecular docking studies were carried out. The docking results showed that GDCA could bind well to FXR, with a docking score of 13.43. The hydrogen bonding between the residues Met265, Met328, Arg331 and GDCA played an important role in the observed interaction (Supplementary Fig. 12a). In addition, the molecular backbone of GDCA is known to have a wide range of hydrophobic interactions with the receptor, facilitating the stabilization of the hydrophobic core of the receptor, which might have acted as a possible direct agonist of FXR in this study (Supplementary Fig. 12b). We further performed a TR-FRET FXR coactivator assay to validate whether GDCA was a direct FXR agonist. Similar to CDCA and GW4064, which were the positive controls, GDCA showed agonistic action (Fig. 4g). We further observed that supplementation of GDCA to *B. fragilis*-treated mice decreased the serum C4 level (Fig. 4h), consistent with previous studies that have shown GDCA can inhibit bile acid synthesis^29,30^. Supplementation with GDCA decreased serum and hepatic TBA levels (Supplementary Fig. 13a; Fig. 4i) and liver injury (Fig. 4j; Supplementary Fig. 13b–e) and increased pup numbers (Supplementary Fig. 13f, g), fetal weight (Supplementary Fig. 13h), placental weight (Supplementary Fig. 13i), and morphological changes in the placenta (Fig. 4j), while deoxycholic acid (DCA) and glycochenodeoxycholic acid (GCDCA) were unable to mitigate and even aggravated *B. fragilis*-induced cholestasis (Supplementary Fig. 14). Furthermore, the expression levels of FXR target gene mRNAs were significantly reversed by GDCA (Fig. 4k, l). Collectively, our data indicate that *B. fragilis* suppressed FXR signaling by modulating bile acid metabolism and induced hepatic bile acid accumulation to promote ICP development.

### FXR signaling is required for *B. fragilis*-induced ICP in mice

**Fig. 5:**
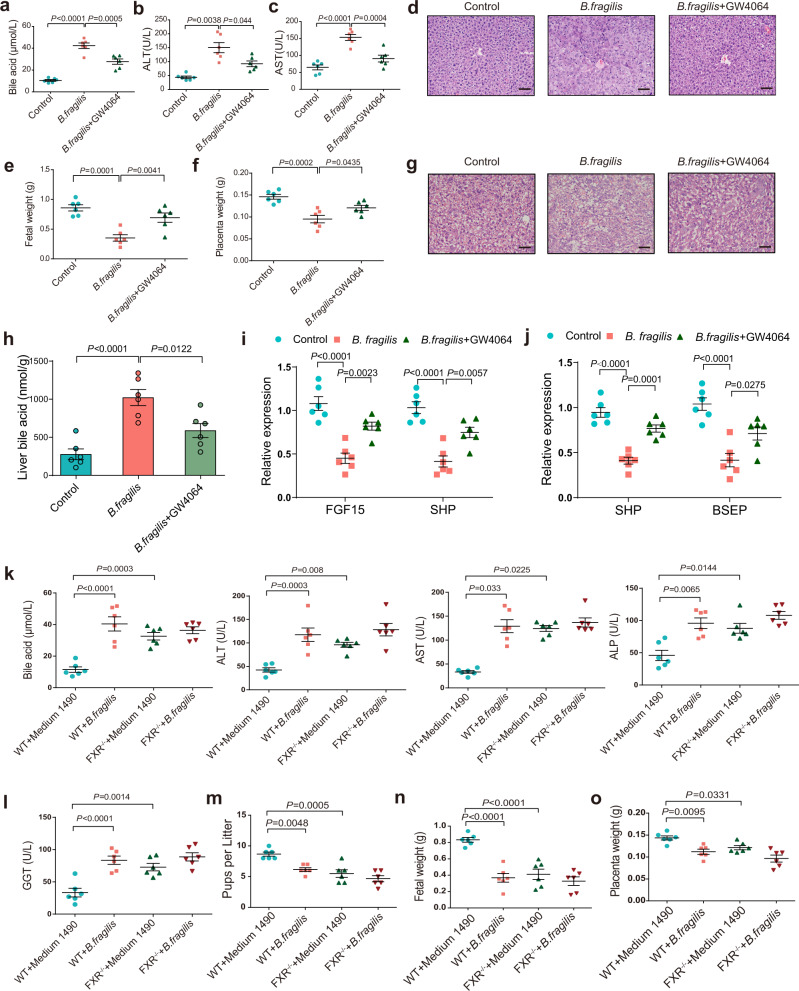
FXR signaling is required for *B. fragilis*-induced ICP in mice. **a**–**c** Analysis of serum levels of total bile acids (**a**), ALT (**b**) and AST (**c**) in each group (*n* = 6 per group). *P* values were determined by one-way ANOVA with Tukey’s correction or Welch ANOVA with Games-Howell’s multiple comparisons test. **d** Representative images of H&E staining of liver in each group. Scale bar: 50 μm. **e**, **f** Fetal weight (**e**) and placenta weight (**f**) in each group (*n* = 6 per group). *P* values were determined by one-way ANOVA with Tukey’s correction. **g** Representative images of H&E staining of placenta in each group. Scale bar: 50 μm. **h** Liver total bile acid levels in each group (*n* = 6 per group). **i**, **j** Relative expression of intestinal FXR target genes (**i**) and hepatic FXR target genes (**j**) in indicated groups. *n* = 6 per group. *P* values were determined by one-way ANOVA with Tukey’s correction. **k**, **l** Serum levels of total bile acids, ALT, AST, ALP, GGT in each group (*n* = 6 per group). **m**–**o** Number of pups per litter (**m**), fetal weight (**n**), and placenta weight (**o**) in each group (*n* = 6 per group). *P* values were determined by one-way ANOVA with Tukey’s correction or Welch ANOVA with Games-Howell’s multiple comparisons test or Kruskal-Wallis with Dunn’s multiple comparisons test depending on the sample distribution type for **h-o**. Data are presented as mean ± SEM for **a**–**c**, **e**, **f**, **h**–**o**. Source data are provided as a Source Data file.

Since our previous data revealed that *B. fragilis*-mediated FXR signaling suppression, we further sought to investigate the role of FXR in *B. fragilis*-induced ICP phenotype. We hypothesized that FXR activation may restore the effects of *B. fragilis*. To test this hypothesis, we used the GW4064, a general FXR agonist, and found that GW4064 could substantially reverse *B. fragilis*-induced cholestasis (Fig. 5a), liver injury (Fig. 5b–d; Supplementary Fig. 15a–d), fetal growth inhibition (Fig. 5e, f; Supplementary Fig. 15e), and morphological changes in the placenta (Fig. 5g).

Moreover, we observed that GW4064 significantly reversed *B. fragilis*-induced hepatic bile acid levels (Fig. 5h). As expected, the reduced expression of FXR target genes in intestine and hepatic tissues were almost completely restored by GW4064 (Fig. 5i, j). Furthermore, *B. fragilis* was gavaged into FXR knockout (FXR^−/−^) mice and wild-type (WT) mice. Consistent with previous results, *B. fragilis* greatly suppressed intestinal and hepatic FXR signaling in WT mice, while it had no effects in FXR^−/−^ mice (Supplementary Fig. 16a, b). *B. fragilis* substantially promoted cholestatic liver injury (Fig. 5k, l; Supplementary Fig. 16c, d), disrupted fetal growth (Fig. 5m, n), and induced placental dysfunction in WT mice (Fig. 5o; Supplementary Fig. 16e), whereas it had no effect in FXR^−/−^ mice because of FXR deletion in these mice. Thus, our data revealed that FXR signaling was required for *B. fragilis*-mediated ICP-like phenotype.

### *B. fragilis* induces excessive bile acid synthesis and inhibits hepatic bile acid excretion through suppression of FXR signaling to promote ICP

**Fig. 6:**
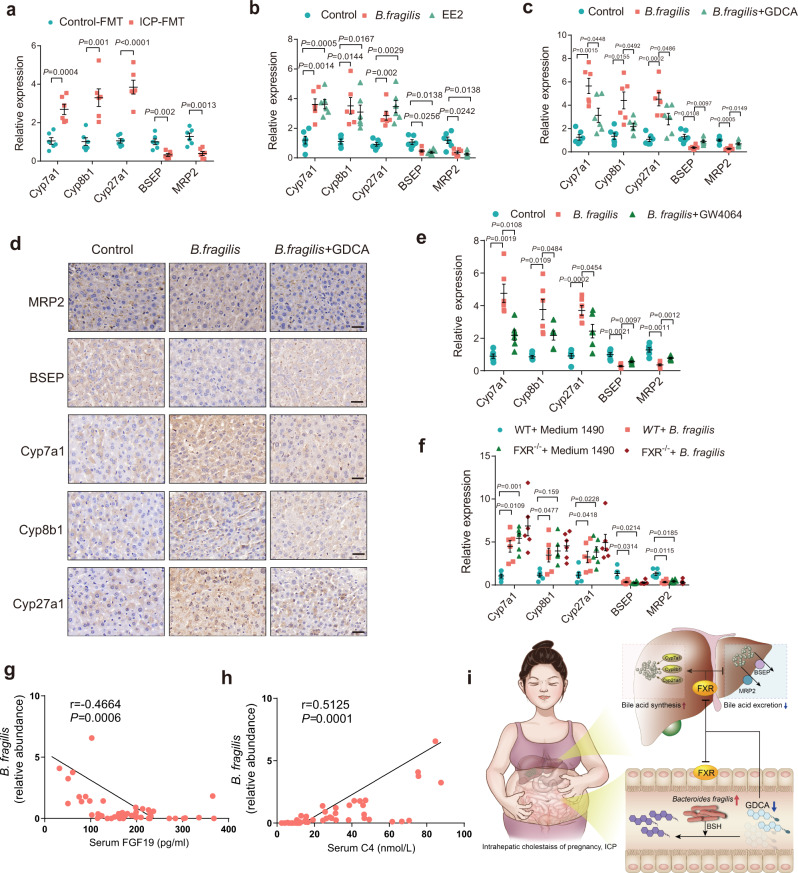
*B. fragilis* induces excessive bile acid synthesis and inhibits hepatic bile acid excretion through suppression of FXR signaling to promote ICP. **a** Hepatic mRNA expression levels of bile acids synthetic and bile excretory genes in mice transplanted with fecal microbiota of ICP and healthy controls (*n* = 6 per group). *P* values were determined by two-tailed Student’s t-test. **b** Hepatic mRNA expression levels of bile acids synthetic and bile excretory genes in control group, *B. fragilis* group and EE2 group (*n* = 6 per group). **c** Hepatic mRNA expression levels of bile acids synthetic and bile excretory genes in each group (*n* = 6 per group). *P* values were determined by Welch ANOVA with Games-Howell’s multiple comparisons test for **b**, **c**. **d** IHC staining of hepatic bile acids synthetic and bile excretory proteins of mice colonized with *B. fragilis* together with GDCA or not. Scale bar: 20 μm. **e** Hepatic mRNA expression levels of bile acids synthetic and bile excretory genes in mice colonized with *B. fragilis* together with GW4064 (10 mg/kg/d) or not (*n* = 6 per group). **f** Hepatic mRNA expression levels of bile acids synthetic and bile excretory genes in WT mice or FXR^−/−^ mice colonized with *B. fragilis* or not (*n* = 6 per group). *P* values were determined by Welch ANOVA with Games-Howell’s multiple comparisons test for **e**, **f**. **g**, **h** Correlations between *B. fragilis* abundance and FGF19 (**g**) or C4 (**h**) were determined by Spearman’s rank test. **i** Schematic mechanisms underlying the role of the *B. fragilis*-bile acid-FXR axis in regulating ICP. Data are presented as mean ± SEM for **a**-**c**, **e**, **f**. Source data are provided as a Source Data file.

The possible mechanisms by which *B. fragilis/*FXR signaling regulates bile acid metabolism and cholestasis in ICP were further investigated. Since cholestasis is mainly characterized by bile flow impairment that mainly involves excessive bile acid synthesis and disrupted hepatic bile excretion, hepatic bile acid synthesis and excretory genes were then measured in different mouse models. Mice transplanted with ICP stool exhibited much higher levels of *Cyp7a1*, *Cyp8b1* and *Cyp27a1* mRNAs and lower expression levels of *BSEP* and *MRP2* than control livers (Fig. 6a). Similarly, we observed that *B. fragilis* induced bile acid synthesis markers and decreased the bile excretory genes *BSEP* and *MRP2* in the liver (Fig. 6b). Notably, the relative mRNA expression levels of *Cyp7a1*, *Cyp8b1*, *Cyp27a1*, *BSEP* and *MRP2* in the liver were dramatically reversed by GDCA administration in *B. fragilis*-treated mice (Fig. 6c). Immunohistochemical staining confirmed that GDCA administration significantly reversed the protein expression levels of the hepatic bile acid synthetic enzymes Cyp7a1, Cyp8b1, Cyp27a1 and the excretory proteins BSEP and MRP2 in the livers of *B. fragilis*-gavaged mice (Fig. 6d). Additionally, modulation of FXR signaling by the agonist GW4064 significantly attenuated *B. fragilis*-induced bile acid synthetic markers and elevated *BSEP* and *MRP2* expression levels (Fig. 6e). We further observed that *B. fragilis* significantly upregulated bile acid synthesis genes expression and suppressed the expression of *BSEP* and *MRP2* in WT mice, whereas no further effect was noted in FXR^−/−^ mice (Fig. 6f). These data demonstrated that *B. fragilis*-mediated FXR signaling inhibition was responsible for excessive bile acid synthesis and interrupted hepatic bile excretion to ultimately promote the initiation of ICP. We further evaluated the relationship among *B. fragilis*, FXR signaling and bile acid synthesis in the ICP cohort. We found that the relative abundance of *B. fragilis* was negatively correlated with the levels of FGF19 (Fig. 6g), but positively related to the concentrations of C4 (Fig. 6h). Together, these clinical data suggest that *B. fragilis* suppressed FXR signaling to increase bile acid synthesis during ICP development, implying a promising intervention target for ICP treatment.

## Discussion

ICP is emerging as a noteworthy pregnancy-related disease that is accompanied by an increased risk of adverse perinatal outcomes. As the mechanism remains poorly understood, therapies have been largely empiric, and there remains an urgent unmet clinical need. Herein, we found that the gut microbiota were changed in patients with ICP and that transplantation of gut microbiota from patients with ICP was sufficient to promote an ICP phenotype in mice. Notably, the microbiomes of patients with ICP were primarily characterized by *B. fragilis*, and *B. fragilis* was markedly increased in patients with severe ICP. *B. fragilis* suppressed FXR signaling by mediating bile acid metabolism through its BSH activity.

Studies have demonstrated that specific alterations in the gut microbiome during pregnancy are closely related to many pregnancy-related diseases, including gestational hypertension, GDM and metabolic syndrome^22–24^. A previous study revealed that normal pregnancy was characterized by a higher ratio of *Bacteroidetes* to *Firmicutes*^25^. Another study also showed that there was notable variation in *Bacteroides* and *Firmicutes* abundances between samples from the first (T1) to third (T3) trimesters^17^. It is possible that the gestational increase in oestrogen, progesterone and corticosteroids exerts microbial selection pressures on the growth of different gut bacteria^31,32^. A previous study reported that the abundance of *Blautia*, *Citrobacter* and *Streptococcus* was significantly higher in ICP patients^27^, and a higher proportion of the genera *Escherichia_Shigella*, *Olsenella*, and *Turicibacter* was observed in patients with severe ICP ^28^. In the present study, we observed a notable difference in microbial profiles between individuals with ICP and healthy controls. We found that the microbiomes of patients with ICP were primarily characterized by *Bacteriodes fragilis*, *Klebsiella pneumoniae*, *Klebsiella variicola*, *Klebsiella quasipneumoniae*, *Weissella confusa*, *Citrobacter youngae*, and *Enterobacter cloacae*, while the microbiomes of patients with severe ICP were mainly characterized by *B. fragilis*. We noticed that there were differences and discrepancies among the previous two studies and our study. We believe the following factors might have contributed to these discrepancies: (1) Previous studies used 16S rRNA sequencing, while we used metagenomic sequencing analysis. (2) Previous studies characterized taxonomic differences through LefSe analysis, while we used MaAsLin2 to analyze the bacterial taxonomic differences. (3) The stool samples for sequencing in the previous studies were from one area, while we recruited patients with ICP and healthy controls from different regions. We believe that there might also be other factors contributing to the discrepancies. Although we did not observe a significant difference in the microbial diversity between the mild and severe ICP groups, *B. fragilis* was notably enriched and increased in abundance in individuals with severe ICP compared to the mild group and was positively correlated with the core features of ICP. It is well known that bile acids and the gut microbiota maintain a complex and bidirectional relationship. Elevated levels of bile acids and *Bacteroidetes* have been found in normal pregnancy with advancing gestation^25^. Previous studies have identified *B. fragilis* as a bile-resistant bacteria ^33^, and have revealed that bile salts can enhance resistance to structurally unrelated antimicrobial agents, bacterial coaggregation, intestinal colonization and biofilm formation of *B. fragilis*^34^. Here, the present study showed that the abundance of *B. fragilis* was increased in patients with ICP and severe ICP. Thus, we believe that there is a possibility that the increased bile acids could cause increases in *B. fragilis* abundance and could enhance intestinal colonization.

Gut microbiota are able to participate in various metabolic processes, including those related to bile acids, short-chain fatty acids (SCFAs), trimethylamine-N-oxide (TMAO), endogenous ethanol, indole, and other metabolites, which are important in intestinal barrier function, the immune system, and host intestinal homeostasis^35–37^. We observed that conjugated bile acid levels were dramatically decreased after faecal transplantation from patients with ICP or after *B. fragilis* transplantation. Bile acid deconjugation is mediated by gut microbes via bile salt hydrolase (BSH) activity. In fact, functional BSH is present in some major bacterial groups, including *Lactobacillus*, *Bifidobacterium*, *Clostridium* and *Bacteroides*^26^. The human and mouse bile acid profiles are known to differ with the predominance of taurine conjugation in mice and glycine conjugation in humans. We observed that the levels of glycine-conjugated bile acids were much lower and that some were undetectable in the mouse small intestine. Bacterial BSH from the faeces of patients with ICP or with *B. fragilis* was able to convert GDCA (in humans) or TDCA (in mice) to DCA. Previous studies have reported that the BSH enzyme has substrate specificity, which might be affected by the optimal pH, substrate concentrations, different monomeric subunits of the enzyme, and so on^38,39^. The BSH enzyme in *B. fragilis* has been found to have the strongest binding affinities for the glycine or taurine conjugates of DCA (GDCA or TDCA) and the weakest binding affinities for glycine or taurine conjugates of CA (GCA or TCA)^38,40^. Moreover, BSH from *B. fragilis* shows lower activity using GCDCA and TCA as substrates and undetectable activity with glycine- or taurine-conjugated LCA as substrates^38^. We also found that *B. fragilis* had lower activity in conjugated CA or CDCA compared to conjugated DCA, further demonstrating the substrate specificity of the BSH enzyme in *B. fragilis*, which might contribute to the differences in bile acid species after *B. fragilis* colonization. Here, we observed that TDCA levels (corresponding to GDCA levels in humans) were much lower in mice transplanted with faeces from patients with ICP or with *B. fragilis* colonization. Although we did not observe a significant difference in DCA levels, there was an increasing trend, which might cause more damage to the liver and promote ICP development. GDCA is nearly undetectable in the mouse small intestine because taurine conjugation is predominant in mice. Since GDCA (corresponding to TDCA in mice) is predominant in humans and *B. fragilis* can deconjugate GDCA with higher activity, we focused on the role of GDCA in *B. fragilis*-induced ICP.

FXR is expressed in the ileum and liver and has been extensively explored for its important role in bile acid homeostasis^41,42^. FXR activation can lead to downregulation of bile acid synthesis, increase bile acid excretion, and decrease bile acid reabsorption^43^. A previous study showed that FXR function was reduced in pregnancy, together with reduced enterohepatic cycling and elevated serum bile acids^25^. We also observed a reduction in intestinal FXR signaling and enterohepatic feedback in patients with ICP and found that intestinal and hepatic FXR signaling was inhibited after *B. fragilis* colonization. We found that *B. fragilis* could cause cholestasis through inhibiting FXR signaling, while *B. fragilis* could not cause worse cholestasis in FXR knockout mice, suggesting that FXR is essential for *B. fragilis*-induced cholestasis. However, the whole body FXR knockout is limited in identifying the specific role of intestinal and hepatic FXR signaling in *B. fragilis*-induced cholestasis. It is known that alterations in the intestinal bile acid composition, resulting from bacterial bile acid modification, can impact enterocyte FXR induction in two ways: by impairing bile acid uptake by the enterocyte and/or by changing the ratio of agonistic to antagonist bile acid ligands^25^. We observed lower levels of conjugated bile acids, with a decrease in T-αMCA, T-βMCA, and TDCA in the mouse small intestine after *B. fragilis* colonization. Since T-αMCA and T-βMCA are FXR antagonists^44^, we hypothesized that the profiles of small intestinal bile acids in *B. fragilis*-colonized mice might not be the primary contributors to the impairment of intestinal FXR function, given that there were reductions in the antagonistic species. We believe that the impairment of intestinal FXR signaling might be attributed to the decrease in conjugated bile acids and reduced terminal ileal uptake because of the lower ASBT affinity of unconjugated bile acids in ileal uptake. Previous studies reported that *B. fragilis* also possessed 7α-hydroxysteroid dehydrogenase (7α-HSDH) activity, which could convert chenodeoxycholic acid (CDCA) into its 7-oxo derivative (7-oxo-lithocholic acid, 7-oxo-LCA)^45,46^. We observed a decreasing trend in CDCA levels in the small intestine of *B. fragilis*-colonized mice, which might further contribute to the reduced intestinal FXR signaling. In addition, we revealed that hepatic levels of T-αMCA and T-βMCA were significantly increased in *B. fragilis*-colonized mice, and given that T-αMCA and T-βMCA have been reported to be FXR antagonists^44^, these increases might contribute to the reduced hepatic FXR signaling.

A previous study reported that DCA could activate FXR, while GDCA or TDCA were inactive against FXR in CHO cells transfected with FXR^47^. Wang et al. reported that the glycine or taurine conjugates of CDCA and DCA could activate FXR in vivo but were inactive in certain cell lines, and further revealed that the conjugated bile acids could not readily cross cell membranes, leading to the inactivity of FXR in cell lines. The researchers cotransfected bile acid transporters in cell lines and observed that the glycine or taurine conjugates of CDCA and DCA were highly effective in activating FXR in cells coexpressing bile acid transporters^48^. Thus, we believe that the different research methods might have caused the discrepancy in the role of GDCA in activating FXR signaling observed in previous studies. In the present study, we applied the TR-FRET FXR coactivator assay and luciferase reporter assay in cells transfected with bile acid transporters to verify that GDCA could activate FXR in vitro. The primary bile acids CA and CDCA and their conjugated counterparts are the major bile acids that are elevated in the serum of patients with ICP, while DCA and GDCA are not the predominant bile acid species. However, we revealed that CA and CDCA or their conjugated counterparts were not changed, while GDCA or TDCA was changed significantly in faecal samples of patients with ICP or in mouse small intestines of ICP faecal transplantation or *B. fragilis* colonization mice. Thus, we focused on bile acid and its role in *B. fragilis*-induced ICP development. We revealed that GDCA administration could activate FXR signaling and alleviate *B. fragilis*-induced cholestasis in vivo. We observed that DCA and GCDCA could not mitigate *B. fragilis*-induced cholestasis and may even aggravate *B. fragilis*-induced cholestasis. Previous studies have reported that DCA is a toxic bile acid that could induce cholestasis, and GCDCA was previously reported to accumulate in human cholestasis and was able to induce the apoptosis of cholangiocytes and hepatocytes^49–53^. In the present study, we found that GDCA activated FXR signaling, inhibited bile acid synthesis, and mitigated *B. fragilis*-induced cholestasis. Since GCDCA was not the target bile acid altered by *B. fragilis* colonization and was able to further aggravate *B. fragilis*-induced cholestasis, we cannot simply say that conjugated bile acids are the key to inhibiting cholestasis induced by *B. fragilis* colonization.

Cholestasis is mainly characterized by bile flow impairment and excessive bile acid accumulation^43,54^. Bile acids are synthesized in the liver and secreted into the intestine through the biliary tract, and are mediated by negative feedback regulation through FXR^42^. CYP7A1, CYP8B1 and CYP27A1 are liver-specific microsomal cytochrome P450 enzymes that act as the rate-limiting step in bile acid synthesis^42^. Additionally, bile acid homeostasis mainly relies on transporters such as the bile salt export pump (BSEP) and multidrug resistance-associated protein 2 (MRP2) to excrete bile acids into the bile ducts^55,56^. In the liver, FXR activation can induce small heterodimer partner (SHP) expression, which then binds to liver receptor homologue-1 (LRH-1) and inhibits *CYP7A1*, *CYP8B1* and *CYP27A1* expression^26,57^. Moreover, FXR activation in the ileum induces FGF15 expression in mice (FGF19 in humans) to bind to FGF receptor 4 (FGFR4) and triggers the JNK1/2 and ERK1/2 signaling cascade, which then inhibits CYP7A1 expression^58^. Various studies have shown that *CYP7A1*, *CYP8B1*, *CYP27A1*, *BSEP* and *MRP2* are direct targets for FXR^59,60^. Our data related to elevated fasting C4 indicated excessive bile acid synthesis in ICP, likely secondary to suppressed FXR signaling by ICP faecal transplantation or *B. fragilis* colonization. We found that hepatic levels of T-αMCA and T-βMCA were significantly increased in *B. fragilis*-colonized mice, which might account for the reduced hepatic FXR signaling. Intestinal bile acid composition alterations might affect intestinal FXR signaling by impairing bile acid uptake or by changing the proportion of agonistic and antagonistic bile acids. Bile acid binding to ASBT is the key step in bile acid uptake from the lumen to ileal enterocytes, and ASBT preferentially binds to conjugated bile acids^25^. Our data revealed significantly reduced conjugated bile acids after *B. fragilis* colonization, which might have reduced intestinal FXR induction secondary to the reduced bile acid uptake. Previous studies showed that activation of FXR by GW4064 protected animals from cholestasis^61,62^. Herein, we provide evidence that administration of either GDCA or GW4064 to activate ileum and hepatic FXR signaling attenuated *B. fragilis*-induced expression of *CYP7A1*, *CYP8B1* and *CYP27A1* and restored expression of *BSEP* and *MRP2*, suggesting that modulation of FXR signaling might be an effective strategy for drug development in ICP.

Currently, the mainstay of treatment for ICP is ursodeoxycholic acid (UDCA), which is produced by the gut microbiota^63^. UDCA is a polar bile acid without a direct FXR agonist effect, and it functions by decreasing the toxicity and hydrophobicity of the bile pool^64,65^. Although some previous studies have revealed that UDCA was effective in ameliorating pruritus and improving liver function in ICP patients^4,7^, the largest randomized controlled clinical trial on this agent, the PITCHES trial, found that there was no evidence that UDCA could reduce adverse perinatal outcomes^64^. A Cochrane database meta-analysis examining treatment with UDCA versus a placebo in ICP found that UDCA administered to patients with ICP probably resulted in a slight reduction in pruritus, with no significant differences in total bile acid levels^6,7^. Because UDCA is not a direct FXR agonist, potent agonists of FXR might be more effective than UDCA in lowering bile acid levels and improving outcomes in ICP patients. Because of the key role of FXR in bile acid synthesis, transport and excretion, the synthetic FXR agonist obeticholic acid (OCA) has been shown to promote bile acid efflux and reduce bile acid synthesis and has shown promising effects in the treatment of cholestatic conditions such as primary biliary cholangitis^66,67^. A previous study also showed that OCA administration could improve fetal hypercholanaemia in an ICP mouse model^68^. Therefore, bile acid modulation currently remains the mainstay of treatment for ICP. Although UDCA treatment of ICP has been shown to reduce maternal bile acid levels and improve liver function, it is not effective in all patients^64^, and a recent trial revealed no benefit for adverse perinatal outcomes^64^, leading to controversy about its use in ICP. Because of the risk of serious liver injury associated with obeticholic acid (Ocaliva), its use is restricted for some patients by the FDA. Thus, it is urgent to develop more effective and safer treatment strategies for ICP. Microbiota-based therapeutics have the potential to become a major therapeutic modality, encompassing those therapies targeting specific pathogens/pathobionts as well as those aiming to strategically restructure a given microbial community in a specific way. There are many microbial therapeutic strategies, including probiotics, faecal microbiota transplantation, those involving administration of metabolites derived from microbial sources or those based on non-bacterial micro-organisms such as bacteriophages^69,70^. Faecal microbiota transplantation has emerged as a treatment for multiple recurrent *Clostridioides difficile* infections (rCDIs) that are nonresponsive to standard therapy approaches recommended in multiple professional society guidelines^71–73^. Notably, the present study demonstrated that *B. fragilis* could suppress FXR signaling and promote ICP development, implying that manipulating the abundances of specific bacteria might be potentially effective. Therefore, although microbial therapy is not yet used in clinical practice for ICP management, it may have benefits and prospects in the future.

In conclusion, we demonstrated that the abundance of *B. fragilis* was substantially increased in individuals with ICP. *B. fragilis* colonization could suppress FXR signaling by mediating bile acid metabolism and result in excessive bile acid synthesis and disrupted bile acid excretion, highlighting the potential role of gut microbiota-bile acid-FXR axis in ICP development (Fig. 6i).

## Methods

### Human samples

The study was approved by the Ethics Committee of Xinqiao Hospital, Army Medical University (Approved No. 2020-146-01). Written informed consents for participating this study were obtained from all participants. The authors affirm that human research participants provided written informed consent for publication of the potentially identifiable medical data included in this article. Participants didn’t receive cash remuneration. ICP was diagnosed according to the Guidelines for diagnosis and treatment of intrahepatic cholestasis of pregnancy from China with the following criteria: unexplainable pruritus; elevated serum bile acids (≥10 μmol/L); no identifiable cause for liver dysfunction; resolution of symptoms and laboratory values postpartum. Exclusion criteria were as follows: preeclampsia, low platelets (HELLP) syndrome, acute fatty liver of pregnancy, active viral hepatitis and primary biliary cirrhosis; patients receiving any antibiotic or probiotics treatment within 1 months; patients with other pregnant complications such as pregnancy diabetes and hypertensive disorders. All pregnant women with ICP were first-visit patients and did not receive any treatment. 50 individuals with ICP and 41 age, BMI and offspring gender matched healthy pregnant women were recruited from Chongqing and Guangdong province of China. There were 30 mild (TBA range 10–39.9 μmol/L) and 20 severe (TBA ≥ 40 μmol/L) ICP patients included. All the characteristics were summarized in Supplementary Tables 1 and 2.

Age, height, body weight, gestation week, birth weight and Apgar score were recorded, and the body mass index (BMI) was calculated. The gestational weeks were strictly matched within 1 week to reduce the impact of gestational week on gut microbiota. Fecal and blood samples were collected after fasting at least 8 h. Fecal samples were stored at −80 °C immediately until further processed. Biochemical parameters were detected by autoanalyzer.

### Animal study

All animal protocols were approved by the Animal Care and Use Committee of the Army Medical University and adhered to the Animal Ethics Statement (Approved No. AMUWEC2020197). C57BL/6 mice were from Vital River Laboratory (Beijing, China) and housed in a standard specific-pathogen-free environment.

For the fecal microbiota transplantation (FMT) study, the donor stools were randomly collected from six ICP patients and six healthy pregnant controls in the third trimester with matched gestational weeks (within 1 week), matched BMI and offspring gender (Supplementary Table 3). The donor stool samples from six ICP or healthy pregnant controls were mixed with saline solution and centrifuged to collect the supernatant. Then, the aliquot of the mixture was gavaged to the recipient mice, respectively. Before stool transplantation, female mice aged 6–8 weeks were administered antibiotics cocktail (vancomycin, 100 mg/kg; neomycin sulfate, metronidazole and ampicillin, 200 mg/kg) intragastrically once daily for five days. Then, 200 μl of the stool suspension from ICP or healthy controls was gavaged to mice twice a week. To avoid contamination from male mice, all the male mice underwent antibiotics treatment and stool transplantation as same as the female mice. Three weeks later, female mice were housed with male mice at a 2:1 ratio, and pregnancy was confirmed by the presence of vaginal spermatozoa. Various tissues and samples were collected at the 18th day of pregnancy (E18d).

*Bacteroides fragilis* (*B. fragilis*) was purchased from American Type Culture Collection (Cat# ATCC25285, ATCC) and cultured in modified chopped meat medium (ATCC-Medium 1490) in a 37 °C anaerobic incubator. Before *B. fragilis* transplantation, we performed a dose-response analysis to determine the optimal dose. We found that the effect could be dose-dependent, and the dose of 2 × 10^8^ colony-forming units (cfu) could exert the maximum response (Supplementary Fig. 17), and we chose this dose for the following experiments. The male and female recipient mice were administered antibiotics cocktail once daily for five days and gavaged with *B. fragilis* at a dose of 2 × 10^8^ cfu per 200 μl sterile PBS twice a week until end of the pregnancy. Gavage of the same dose of heat-killed *B. fragilis* was used as a negative control. Additionally, the recipient mice were gavaged with *B. fragilis* with or without caffeic acid phenethyl ester (CAPE) (75 mg/kg/d, Cat# HY-N0274, MCE) for 6 weeks to explore the role of BSH activity of *B. fragilis*. To evaluate the S1PR2 activation in *B. fragilis* colonized mice, the recipient mice were gavaged with *B. fragilis* with or without JTE-013 (Cat# HY-100675, MCE) (S1PR2 antagonist, 40 mg/kg/d). To evaluate the effect of GDCA in *B. fragilis*-induced ICP, the recipient mice were gavaged with *B. fragilis* with or without GDCA (30 mg/kg/d; once a day for 3 weeks) (Cat# IG2290, Solarbio). Furthermore, mice gavaging *B. fragilis* were administered intragastrically with GW4064 (10 mg/kg/d, Cat# HY-50108, MCE) to explore the role of FXR. Experimental intrahepatic cholestasis of pregnancy is induced by subcutaneous injections of 17α-ethynylestradiol (EE2) (5 mg/kg, Cat# E4876, Sigma-Aldrich) once daily for 6 days from the 12th day of pregnancy (E12d). The control group was injected daily with corn oil. B6.129 × 1(FVB)-*Nr1h4*^tm1Gonz^/J (FXR^−/−^) mice were obtained from Jackson Laboratory (Cat# 00724). Six- to eight-week-old female FXR^−/−^ mice and wild type (WT) mice were gavaged with *B. fragilis* (2 × 10^8^ cfu/200 μl) or same dose of heat-killed *B. fragilis*. To avoid contamination from male mice, all the male mice underwent antibiotics treatment and *B. fragilis* transplantation as same as the female mice.

Before the mice were killed, the animals from each group were measured weight. The serum was collected after 4 h fasting. Livers, placenta, fetal were weighed and the livers and placenta tissues were further processed for hematoxylin and eosin (HE) staining or immumohistochemical staining. Feces and intestines were stored at −80 °C for further use.

### DNA extraction and 16S rRNA sequencing

Fecal bacterial DNA was extracted using TIANamp Stool DNA Kit (Cat# DP328, TIANGEN Biotech Co. Ltd., China) according to the manufacture’s instruction. The quantity and quality were measured using a Nanodrop (Thermo Scientific, USA). The V3-V4 hypervariable regions of the 16S rRNA were amplified. PCR amplicons were purified and sequenced on the Illumina HiSeq platform (Illumina, San Diego, USA) by Magigene (Magigene Guangzhou, China). The Quantitative Insights into Microbial Ecology 2 (QIIME2, version 2019. 7) platform was used to process the sequencing data. The V3-V4 primers of pair-end fastq format sequence files were trimmed by using cutadapt 3.1 with Python 3.6.9 and imported into QIIME2. The beta diversity was calculated by “qiime diversity beta” command from the rarefied feature-table. The PCoA results were calculated by “qiime diversity pcoa” command and visualized by “qiime emperor plot” command.

### Metagenomic sequencing and analysis

Total microbial DNA were extracted using the QIAamp PowerFecal Pro DNA Kit (Cat#51804, QIAGEN). DNA concentration was measured. 1 μg DNA per sample was used as input. Sequencing libraries were generated using NEBNext® Ultra™ DNA Library Prep Kit (Cat# E7370L, NEB). DNA samples were fragmented by sonication to 350 bp, which were end-polished, A-tailed, and ligated. PCR products were purified. The clustering of the index-coded samples was performed on a cBot Cluster Generation System, and then sequenced on an Illumina Novaseq 6000 platform by Novogene (Novogene Tianjin, China).

QC process including trimming of low-quality bases, masking of human DNA contamination, and removal of duplicated reads were performed by using kneaddata (version v0.6.1). Human DNA contamination was identified by aligning all raw reads to the human reference genome (hg19) using bowtie2 (version 2.3.5.1). Taxonomic annotation of metagenome and the abundance quantification were performed by MetaPhlAn (version 2.0). Relative abundance of each clade was calculated at six levels (L2: phylum, L3: class, L4: order, L5: family, L6: genus, L7: species). Functional annotations were performed by using the data files from the HMP Unified Metabolic Analysis Network 3.0 (HUMAnN 3.0)^74^. The clean paired-end sequencing data were merged into a single fastq file. The HUMAnN 3.0 toolkit was run by using the “humann–input myseq*.fq–output humann3/–threads 32–memory-use maximum -r -v” command, which calls Bowtie2^75^ to compare nucleic acid sequence and calls DIAMOND^76^ to compare protein sequences to complete gene and protein function annotation to obtain KEGG pathway annotation. Differences in bacterial abundance and functional pathway were analyzed using MaAslin2^77^. Richness indices were calculated using the R Community Ecology Package vegan. Weighted Unifrac distance was calculated using Metaphlan3 R script “Unifrac_distance.r” and root-tree file “mpa_v30_CHOCOPhlAn_201901_species_tree.nwk”. The PCoA results were calculated and visualized using R build-in functions and the plot3D R package. The ANOSIM test was used to calculate the significance of dissimilarity using the R Community Ecology Package vegan. Pearson correlation and *P* values were evaluated using the rcorr function in the Hmisc R package.

### Cell culture

Caco-2 (Cat# HTB-37, ATCC) and HEK293 cells (Cat#CRL-3216, ATCC) were purchased from ATCC (Manassas, VA) and cultured in DMEM with 10% FBS. Cells were then exposed to different concentrations of specific bile acids for 24 h. HEK293 cells were transfected with different vectors and used for luciferase reporter assay.

### Bile acid analysis

50 μL of serum was mixed with 150 μL of methanol. The mixture was vortexed for 2 min and centrifuged at 20,000 × *g* at 4 °C for 10 min. 160 μL of supernatant was vacuum-dried. The residue was redissolved with acetonitrile and water to a volume of 40 μL. Supernatant was used for UPLC-MS/MS analysis. 200 μL aliquot of methanol/water (1:1) was added to 10 mg of fecal samples. Samples were homogenized and centrifuged at 13,000 × *g* for 15 min. The supernatant was transferred into a tube and sample residue was extracted by methanol/acetonitrile (2:8). The extraction mixture was vortexed and centrifuged at 13,000 × *g* for 15 min. The supernatant was then used for further analysis.

Bile acid analysis was performed on the UPLC-MS/MS (Waters Corp., USA). The elution solvents were water + 0.01% formic acid (A) and acetonitrile/methanol (19:1) + 0.01% formic acid (B). The elution gradient at a flow rate of 450 μL/min was as follows: 0–2 min (20% B), 2–3 min (20–25% B), 3–6 min (25% B), 6–8 min (25–35% B), 8–11.5 min (35% B), 11.5–18 min (35–99% B), 18–19 min (99% B), and 19–20 min (99–20% B). The peak annotation and quantitation was performed by TargetLynx application manager. Multi Quant 2.1 software were used for bile acids data collection.

### BSH activity analysis

*B. fragilis* proteins were prepared using sonication. The incubation was carried out in 3 mM sodium acetate buffer containing 0.1 mg/ml protein and 0.1 mM d4-GDCA (Sigma-Aldrich, Cat#330271 W, 100 μg) with or without CAPE (MCE, Cat# HY-N0274, 10 mg). The mixtures were incubated at 37 °C and the reactions were stopped in dry ice. 100 μL of methanol was added and the mixtures were vortexed and centrifuged for 20 min. The supernatants were used for d4-GDCA quantification by UPLC-TQMS (Waters, Milford, MA, USA). The BSH activities were predicted by hydrolysis of d4-GDCA. For BSH activity assay in different conjugated bile acids, the mixture contained 2 mL 0.1 M phosphate buffer, 2 mg *B. fragilis* cells, and 500 μg of conjugated bile acids (GDCA, Cat#IG2290; TDCA, Cat#YS167267; GCDCA, Cat#YS175025; TCDCA, Cat#YZ110846; TCA, Cat#T8510; Solarbio) (GCA, Cat#475-31-0, Aladdin). The mixtures were incubated at 37 °C for up to 120 min. The reaction was terminated by adding 200 μL 15% (w/v) trichloroacetic acid. The mixtures were centrifuged at 15,000 × *g* for 10 min to obtain the reaction samples. The deconjugated bile acids were detected using the UPLC-MS/MS (Waters Corp., USA).

### Molecular docking

The crystal structures of the complex of farnesoid X receptor (FXR) and GW 4064 were downloaded from RCSB Protein Data Bank (PDB ID: 3dct, https://www.rcsb.org/) and prepared by SYBYL-X 2.0. The docking analysis was performed using the Surflex-Dock GeomX (SFXC) in SYBYL-X 2.0. The binding interaction was generated using PyMOL and ligplot.

### TR-FRET FXR coactivator assay

Direct FXR activity was evaluated using the LanthaScreen™ TR-FRET Farnesoid X Receptor Coactivator Assay kit (Cat# PV4833, ThermoFisher Scientific). Briefly, prepare a 12-point 100× dilution series of GDCA (Cat#IG2290, Solarbio), CDCA (Cat#IC0300, Solarbio), GW4064 (Cat# HY-50108, MCE) and GUDCA (Cat#IG0840, Solarbio) in a 96-well plate by serial dilution, respectively. Dilute each 100× serial dilution to 2× using Complete Coregulator buffer G. Then, the 2× serial dilutions were mixed with FXR-LBD-glutathione S-transferase fusion protein, fluorecein-SRC2-2 coactivator peptide and Lantha-screen Tb anti-GST antibody (Cat# PV4833, ThermoFisher Scientific, 1:1500) in the 384-well assay plate. Mix the 384-well plate and the TR-FRET signal was evaluated in a Multi-Mode Microplate Reader (Varioskan Flash, Thermo Fisher). Calculate the TR-FRET ratio by dividing the emission signal at 520 nm by the emission signal at 495 nm. Generate a binding curve by plotting the emission ratio vs. [ligand].

### Luciferase reporter assay

pGL3-Basic-SHP firefly luciferase reporter vector, human FXR expression vector, and human ASBT expression vector were constructed by Sangon Biotech (Shanghai, China). HEK293 cells were cultured and co-transfected with human FXR expression vector, human ASBT expression vector, pGL3-Basic-SHP firefly luciferase reporter vector and the Renilla luciferase control vector (Promega, Madison, WI) using Lipofectamine^TM^ 3000 transfection reagent (Cat# L3000015, ThermoFisher Scientific). Luciferase assays were performed by Dual-Luciferase® Reporter Assay System (Cat# E1910, Promega), and Firefly and Renilla luciferase activities were measured by Microplate Reader (Varioskan Flash, Thermo Fisher).

### Measurement of FGF15, FGF19 and C4 in serum

Serum FGF15 levels were tested by the ELISA Kit (Cat# LS-F35359, LifeSpan BioSciences). Briefly, add 100 μL of standards, blank or samples to each well which was pre-coated with FGF15 antibody, and incubate for 90 min at 37 °C. Remove liquid and tap against clean absorbent paper for three times. Add 100 μL of biotinylated detection antibody solution to each well, and gently agitate to ensure thorough mixing. Total mixture was incubated at 37 °C, followed by wash buffer per well for three times. 100 μL of HRP-Streptavidin conjugate working solution was added to each well, incubate for 30 min at 37 °C and washed five times. 90 μL of TMB substrate was added to each well and incubated for 30 min at 37 °C. 50 μL of stop solution was added to each well. Determine the optical density (OD value) of each well using a microplate reader set to 450 nm. The standard stock solution was prepared to generate a standard curve.

Human serum FGF19 levels were quantified using the Human FGF19 SimpleStep ELISA® Kit (Cat#ab230943, Abcam). Briefly, add 50 µL of all samples or standard dilution series to each well and 50 µL of the Antibody Cocktail was added. Incubate the mixture for 1 h at room temperature. Wash each well with wash buffer. 100 µL of TMB solution was added to each well and incubate for 10 min. 100 µL of stop solution was added to each well. Record the OD at 450 nm, create a standard curve, and determine the concentration using the standard curve.

The level of serum 7α-hydroxy-4-cholesten-3-one (C4) was tested by a mass spectrometry-based (MS-based) method. Briefly, serum sample was mixed with acetonitrile containing 1 μM chlorpropamide. Sample injection and flow rate were set at 2 μL and 0.35 ml/min. The samples were separated using an ACQUITY BEH C18 column (1.7 μm, 100 mm × 2.1 mm) with a linear gradient of 0.1% formic acid (FA) in water (A) and 0.1% FA in acetonitrile (B). The eluate delivered into a 5600 TripleTOF (SCIEX, Framingham, MA).

### Real-time quantitative PCR

The tissues were homogenized and total RNA was isolated by Trizol Reagent (Invitrogen, USA). Concentration was measured using the NanoDrop (Thermo Fisher Scientific, USA). Real-time qPCR was performed using the ABI 7500 real-time PCR system (Applied Biosystems). Specific primers for quantitative PCR used in this study are shown in Supplementary Tables 4 and 5. The relative count of genes was calculated by normalizing to 18 S mRNA.

### Western blot analysis

Liver tissues of the mice with different treatment were harvested and lysed with the lysis buffer, respectively. The lysate was kept on ice, followed by the centrifugation at 13,400 × *g* at 4 °C for 20 min. The protein concentration was measured by the BCA assay (Cat# P0012, Beyotime). The protein samples were separated by 10% SDS-PAGE, and then electronically transferred onto the PVDF membrane (Cat#IPVH00010, Millipore). After blocking with 5% non-fat milk at room temperature, the membrane was incubated with the primary antibodies for p-ERK (Cat#4370, Cell Signaling, 1:1000), ERK (Cat#4695, Cell Signaling, 1:1000), p-AKT (Cat#4060, Cell Signaling, 1:1000), and AKT (Cat#9272, Cell Signaling, 1:1000) at 4 °C overnight. After washing, the membrane was incubated with the secondary antibody conjugated with horseradish peroxidase (Cat# A0208, Beyotime, 1: 2000) at 37 °C for 1 h. After the exposure development, the protein bands were imaged and analyzed.

### Histological analysis and immunohistochemistry

Liver and placenta tissues were fixed in 4% paraformaldehyde, dehydrated and embedded in paraffin. The tissues were serially sectioned into 4 μm sections, and stained with hematoxylin and eosin. All sections were mounted onto a glass slide and observed under the light microscope and collected by NIS-Elements 3.2 (Nikon, Tokyo, Japan). Sections were examined by a qualified and blinded pathologist to evaluate the pathological changes.

Tissue sections were deparaffinized and rehydrated using ethanol and distilled water, and treated with 3% H_2_O_2_. Sections were then rinsed twice and incubated with goat serum to block non-specific antibody binding. Immunohistochemistry was performed using the primary antibodies for Cyp7a1 (Cat#bs-21430R, Bioss, 1:100), Cyp8b1 (Cat#bs-14165R, Bioss, 1:100), Cyp27a1 (Cat#bs-5049R, Bioss, 1:100), MRP2 (Cat#bs-1092R, Bioss, 1:100) and BSEP (Cat#bs-12440R, Bioss, 1:100). After washing, sections were incubated with the secondary antibody (PV-6001; Zhongshan, China) for 30 min. The sections were stained with DAB, dehydrated with ethanol and xylene, and then sealed. The slides were photographed using a digital microscope camera and collected by NIS-Elements 3.2 (Nikon, Tokyo, Japan).

### Serum biochemical analysis

Mice blood samples were collected and centrifuged at 3000 × *g* for 10 min, the serum was then collected for the analysis. Serum levels of aspartate transaminase (AST) (Cat#C010-2-1), alanine aminotransferase (ALT) (Cat#C009-2-1), alkaline phosphatase (ALP) (Cat#A059-2-2), gamma-glutamyl transpeptidase (GGT) (Cat#C017-2-1) and total bile acid (TBA) (Cat#E003-2-1) were analyzed by commercially available kits (Jiancheng Institute of Biotechnology, Nanjing, China).

### Statistical analysis

Data were shown as the mean ± S.E.M. The statistical data were collected with Microsoft Excel (2013) and GraphPad Prism software (v9, GraphPad Software Inc., San Diego, USA). The sample distribution was determined by the Kolmogorov-Smirnov normality test. A two-tailed Student’s *t*-test was used to evaluate statistical significance between two groups for normal distribution. For the nonparametric tests, the two-tailed Mann-Whitney test was used to evaluate statistical significance between two groups. One-way analysis of variance (ANOVA) followed by Tukey’s correction was used to evaluate the statistical significance of differences among multiple groups with assumed equal variances. Brown-Forsythe test was used to test homoscedasticity. Welch ANOVA with Games-Howell’s multiple comparisons test was used to evaluate the statistical significance of differences among three or more groups if equal variances were not assumed. For the nonparametric tests among three or more groups, the significance was calculated by Kruskal-Wallis with Dunn’s multiple comparisons test. Correlation analysis was performed using Spearman’s rank test or Pearson correlation analysis. Multiple testing was corrected using the Benjamini-Hochberg method to control the false-discovery rate (FDR). *P* value or FDR-corrected *P* value < 0.05 was considered statistically significant. All data shown were representative results from at least three independent experiments.

### Reporting summary

Further information on research design is available in the Nature Portfolio Reporting Summary linked to this article.

## Supplementary information

Supplementary Information

Reporting Summary

## Data Availability

The raw sequencing data generated in this study have been deposited in the Genome Sequence Archive^78^ in National Genomics Data Center^79^, China National Center for Bioinformation/Beijing Institute of Genomics, Chinese Academy of Sciences (GSA: CRA009840) that are publicly accessible at [https://ngdc.cncb.ac.cn/gsa]. The raw clinical data of participants are protected and are not available due to data privacy laws. All other data supporting the findings generated in this study are provided in the Supplementary Information and Source Data file. Source data are provided with this paper.

## Source data

Source data
