# Supplementary material for: Gut microbiota alters host bile acid metabolism to contribute to intrahepatic cholestasis of pregnancy
Source: Nat Commun. 2023 Mar 9;14:1305. doi: 10.1038/s41467-023-36981-4 (PMC9998625; doi:10.1038/s41467-023-36981-4)
Supplement: Supplementary file 1 — Supplementary Information [file 41467_2023_36981_MOESM1_ESM.pdf]

Gut microbiota alters host bile acid metabolism to contribute to intrahepatic cholestasis of pregnancy

Bo Tang[1,9], Li Tang[1,9], Shengpeng Li[1], Shuang Liu[1], Jialin He[1], Pan Li[2], Sumin Wang[1], Min Yang[1], Longhui Zhang[3], Yuanyuan Lei[1], Dianji Tu[4], Xuefeng Tang[5], Hua Hu[3], Qin Ouyang[6], Xia Chen[2,10], Shiming Yang[1,7,8,10]

1 Department of Gastroenterology, Xinqiao Hospital, Army Medical University, Chongqing, China

2 Department of Obstetrics and Gynecology, First People's Hospital of Foshan, Foshan, Guangdong, China

3 Department of Obstetrics and Gynecology, Xinqiao Hospital, Army Medical University, Chongqing, China

4 Laboratory Medicine Center, Xinqiao Hospital, Army Medical University, Chongqing, China

5 Department of Pathology, Xinqiao Hospital, Army Medical University, Chongqing, China

6 College of Pharmacy, Army Medical University, Chongqing, China

7 Chongqing Institute for Brain and Intelligence, Guangyang Bay Laboratory, Chongqing, China

8 Chongqing Municipality Clinical Research Center for Gastroenterology, Chongqing, China

9 These authors contributed equally

10 These authors jointly supervised this work

Authors for correspondence:

Prof. Xia Chen, MD, PhD.

Department of Obstetrics and Gynecology, First People's Hospital of Foshan, Foshan, Guangdong 510515, China; E-mail: 2676832333@qq.com

Prof. Shiming Yang, MD, PhD.

Department of Gastroenterology, Xinqiao Hospital, Army Medical University, Chongqing 400037, China. E-mail: Yangshiming@tmmu.edu.cn

Supplementary Figure Legends

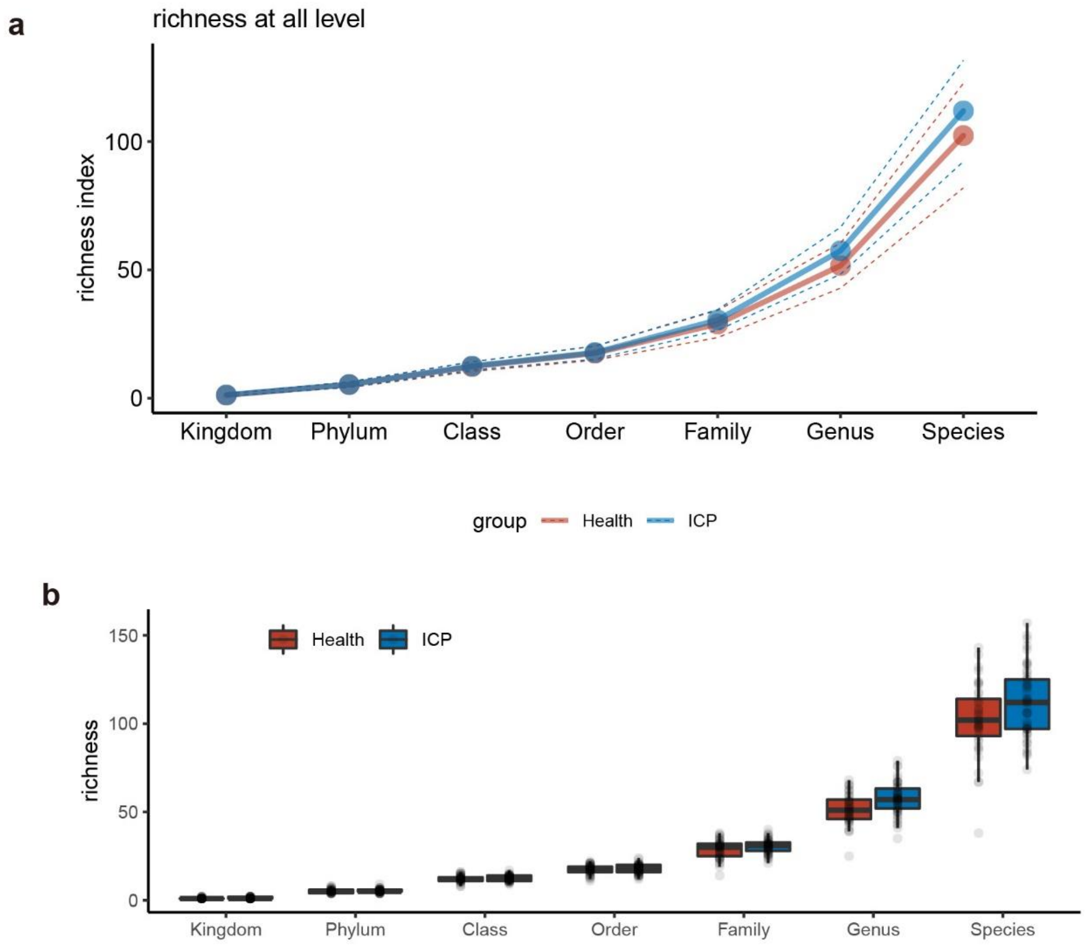

Supplementary Fig.1 a, b Comparison of alpha diversity (richness index) in healthy control and ICP patients. n=50 individuals with ICP, n=41 individuals in healthy controls. The error band in **a** represents the line connecting the standard deviation of each level. For the box plots in **b**, the horizontal bar within box represents median. The top and bottom of box represent 75th and 25th quartiles, respectively. The upper and lower whiskers extended 1.5× the interquartile range from the upper edge and lower edge of the box represent maximum and minimum, respectively.

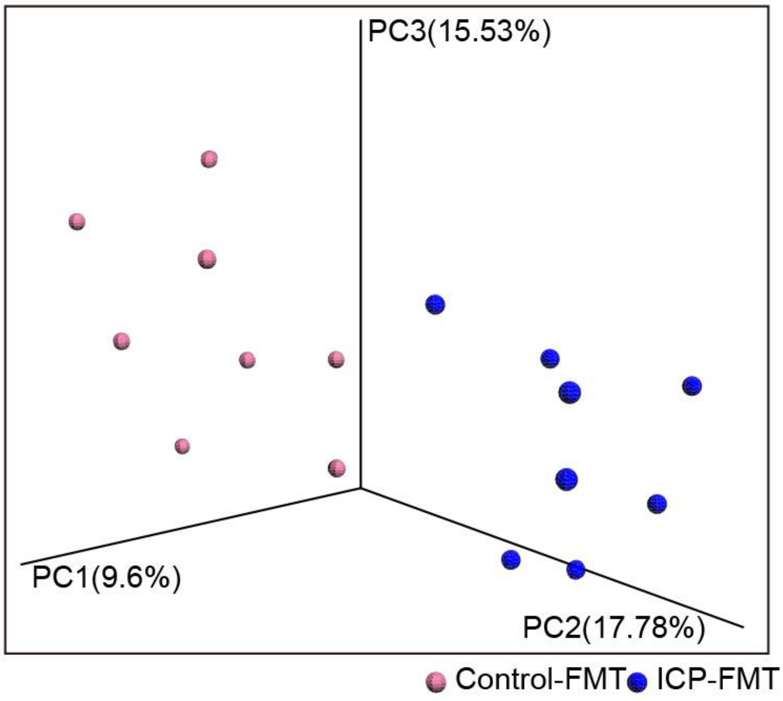

Supplementary Fig.2 Weighted Unifrac PCoA plot of 16S rRNA sequencing from mice transplanted with fecal samples of healthy controls or ICP patients. n=8 per group.

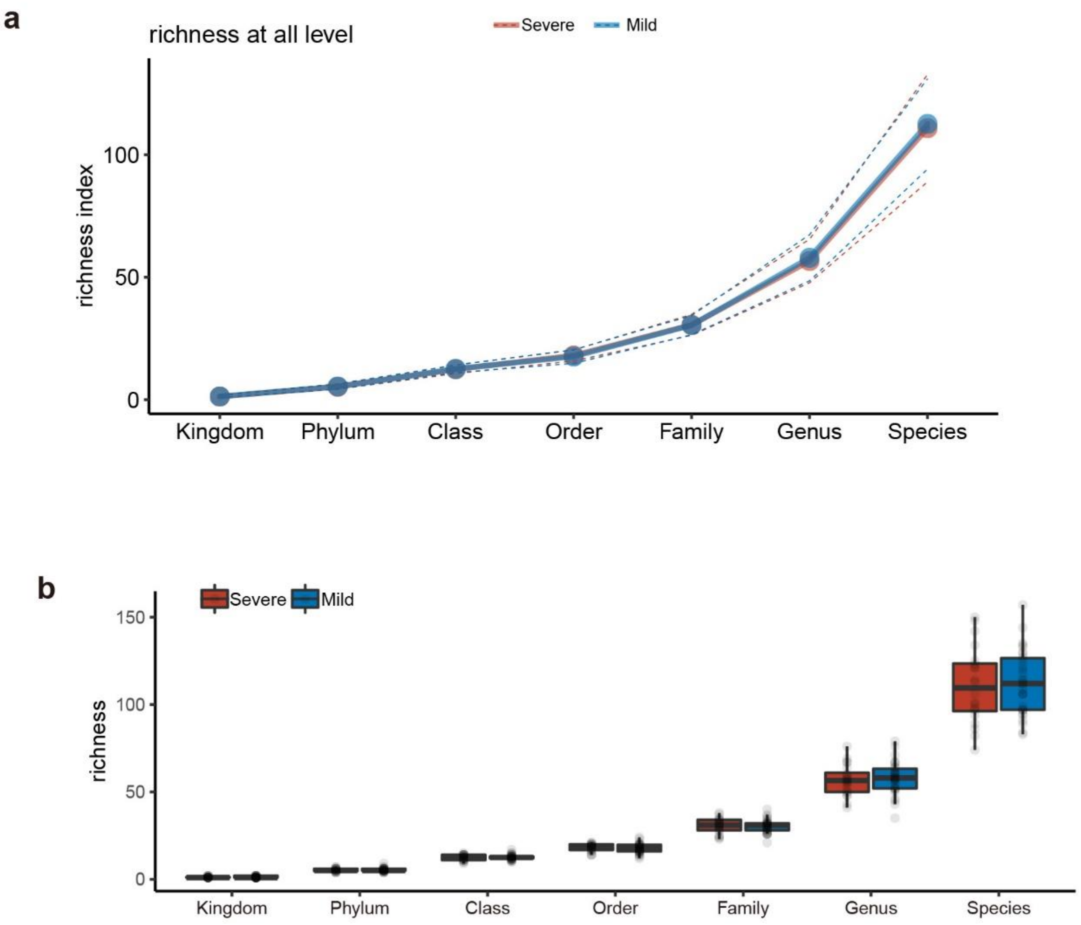

Supplementary Fig.3 a, b Comparison of alpha diversity (richness index) in mild and severe ICP patients. n=20 patients in severe group, n=30 patients in mild group. The error band in **a** represents the line connecting the standard deviation of each level. For the box plots in **b**, the horizontal bar within box represents median. The top and bottom of box represent 75th and 25th quartiles, respectively. The upper and lower whiskers extended 1.5× the interquartile range from the upper edge and lower edge of the box represent maximum and minimum, respectively.

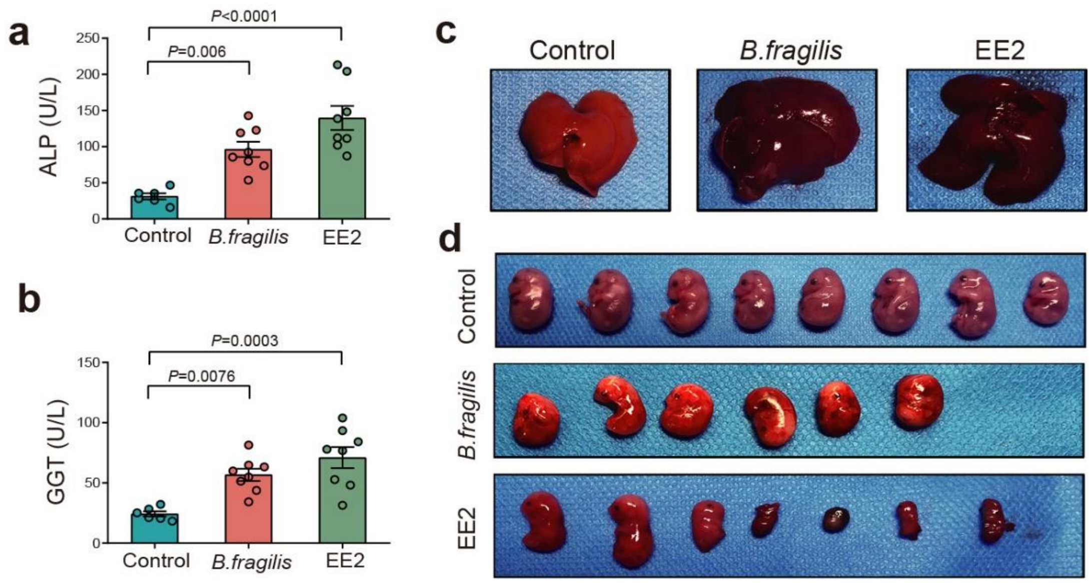

Supplementary Fig.4 ***B. fragilis*** **promotes cholestatic liver injury and disturbs fetal growth in ICP mice model. a, b** Serum levels of ALP (**a**) and GGT (**b**). (n=6 in control group; n=8 in *B. fragilis* group; n=8 in ICP group). Data are presented as mean ± SEM. *P* values were determined by one-way ANOVA with Tukey's correction. **c** Representative images of livers in each group. **d** Representative images of pups per litter in each group. (n=6 in control group; n=8 in *B. fragilis* group; n=8 in ICP group). Source data are provided as a Source Data file.

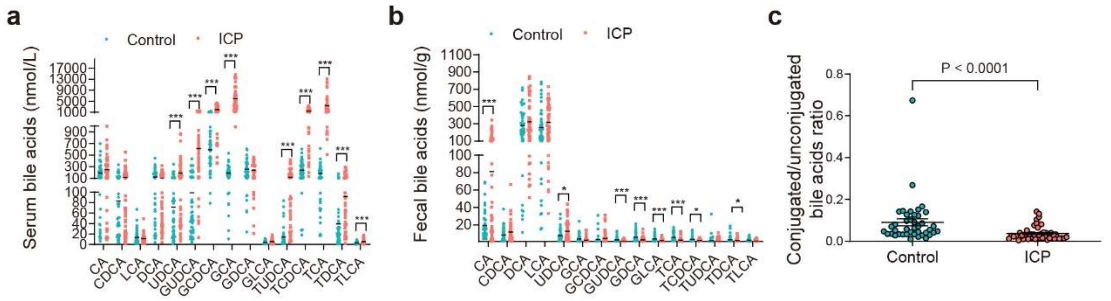

Supplementary Fig.5 Levels of serum and fecal bile acids in ICP patients and healthy controls. Bile acid levels in the serum (**a**) and faeces (**b**) of ICP patients (n=50) and healthy controls (n=41). *P* value was determined by two-tailed Mann-Whitney test. **P*<0.05; ****P*<0.001. **c** The ratio of conjugated to unconjugated bile acids in the faeces of ICP patients and healthy controls. Data are presented as mean ± SEM for **a**-**c**. *P* values were determined by two-tailed Mann-Whitney test. Source data are provided as a Source Data file.

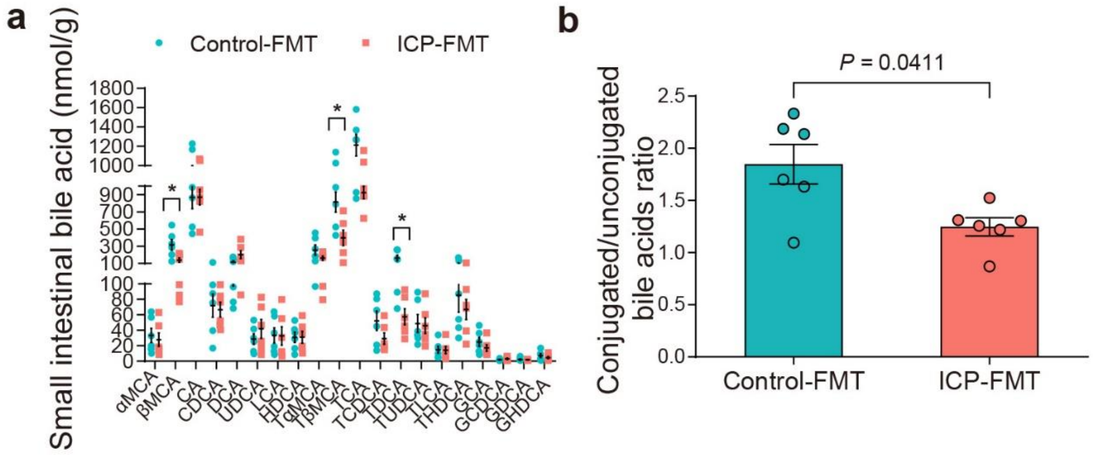

Supplementary Fig.6 Levels of small intestinal bile acids in recipient mice transplanted with ICP stool sample or control stool. a Levels of small intestinal bile acids in recipient mice transplanted with ICP stool sample or control stool (n=6 per group). *P* values were determined by two-tailed Mann-Whitney U test. **P*<0.05. **b** The ratio of conjugated to unconjugated bile acids in ICP fecal transplanted or control stool transplanted mice (n=6 per group). Data are presented as mean ± SEM for **a**, **b**. *P* values were determined by two-tailed Mann-Whitney U test. Source data are provided as a Source Data file.

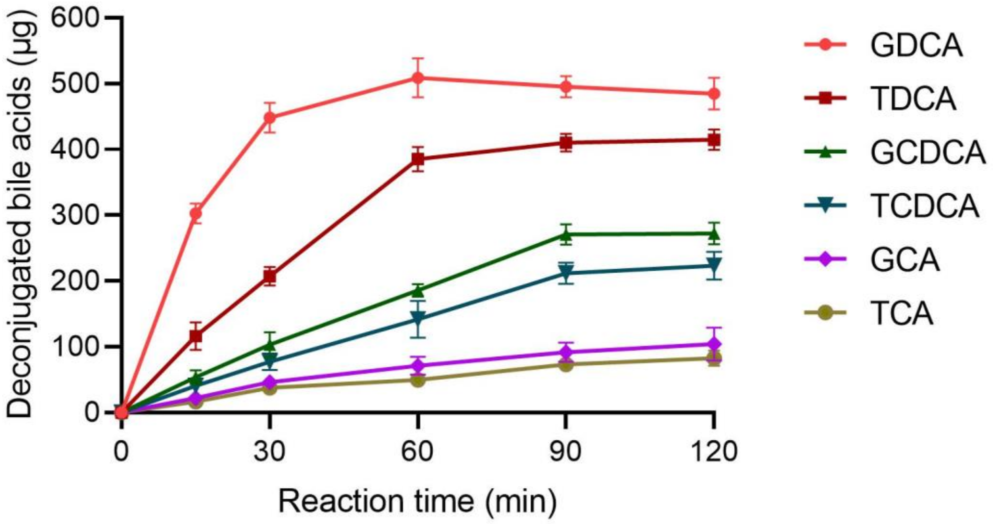

Supplementary Fig.7 Deconjugation of different bile acids by *B. fragilis* in a time-dependent manner. The deconjugated bile acids were detected using the UPLC-MS/MS. Data are presented as mean ± SEM. n=3 per group. Source data are provided as a Source Data file.

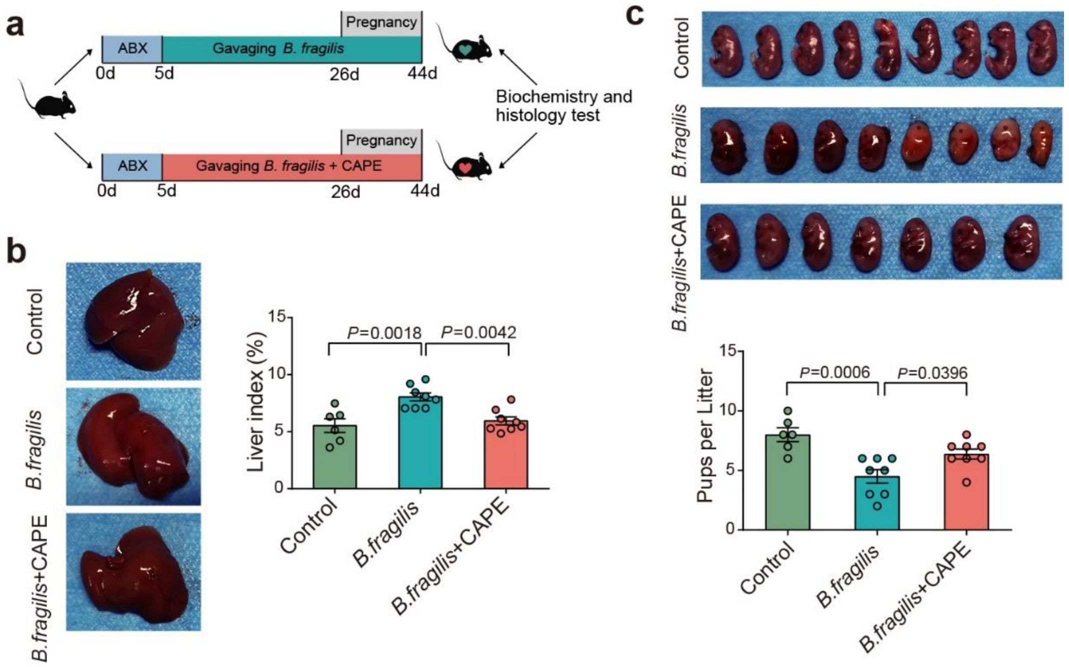

Supplementary Fig.8 CAPE restores *B. fragilis*-induced intrahepatic cholestatic liver injury and fetal dysfunction in pregnancy. a Experimental schematic: following antibiotics treatment, female recipient mice were gavaged with *B. fragilis* at a dose of 2×10^8 colony-forming units (cfu) per 200 µl sterile PBS twice a week until end of the pregnancy with or without CAPE (75 mg/kg/d). Various tissues and samples were collected at E18d. **b** Representative images of livers and liver index in each group (n=6 in control group; n=8 in *B. fragilis* group and *B. fragilis* + CAPE group). Data are presented as mean ± SEM. **c** Representative images of fetuses and number of pups per litter in each group (n=6 in control group; n=8 in *B. fragilis* group and *B. fragilis* + CAPE group). Data are presented as mean ± SEM. *P* values were determined by one-way ANOVA with Tukey's correction. Source data are provided as a Source Data file.

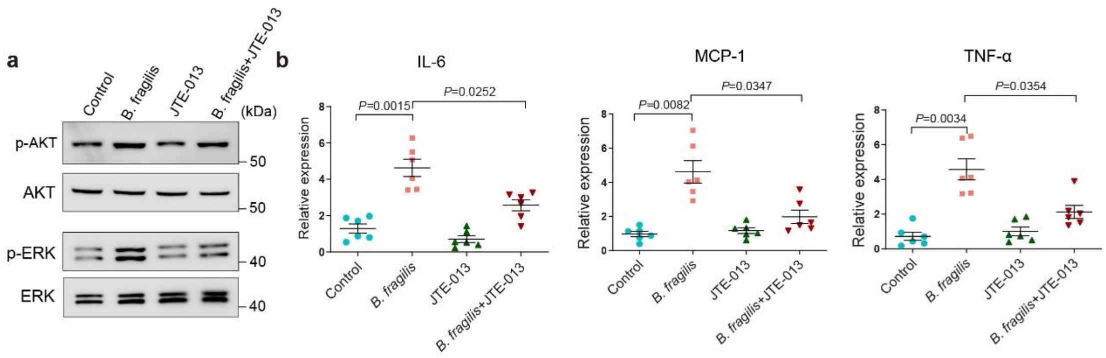

Supplementary Fig.9 *B. fragilis* activated S1PR2 and promoted inflammation in the liver. a The protein levels of phosphorylated AKT (p-AKT), total AKT, phosphorylated ERK (p-ERK) and total ERK in the liver of mice with *B. fragilis* colonization or S1PR2 inhibitor JTE-013 were determined by Western blot analysis. **b** The mRNA levels of IL-6, MCP-1 and TNF-α in the liver of mice with *B. fragilis* colonization or S1PR2 inhibitor JTE-013 were determined by real time RT-PCR. Data are presented as mean ± SEM. n=6 per group. *P* values were determined by Welch ANOVA with Games-Howell's multiple comparisons test. Source data are provided as a Source Data file.

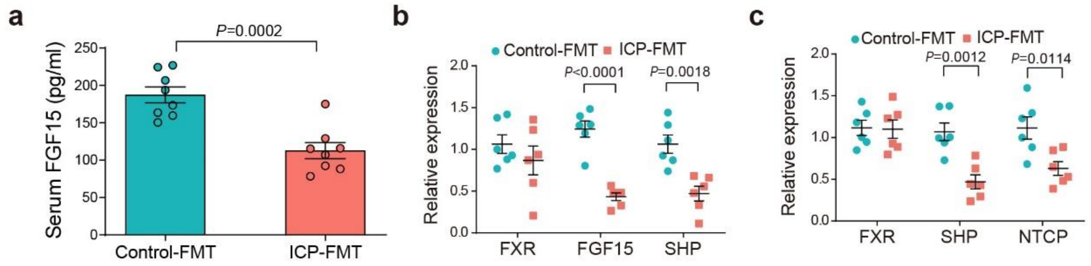

Supplementary Fig.10 ICP microbiota transplantation suppressed the FXR signaling. a Serum FGF15 levels in mice transplanted with fecal microbiota of individuals with ICP or healthy controls. n= 8 per group. *P* values were determined by two-tailed Student's t-test. **b, c** Relative expression of intestinal FXR mRNA and its target genes, and relative expression of hepatic FXR mRNA and its target genes in mice transplanted with fecal microbiota of individuals with ICP or healthy controls. n= 6 per group. *P* values were determined by two-tailed Student's t-test. Data are presented as mean ± SEM for **a**-**c**. Source data are provided as a Source Data file.

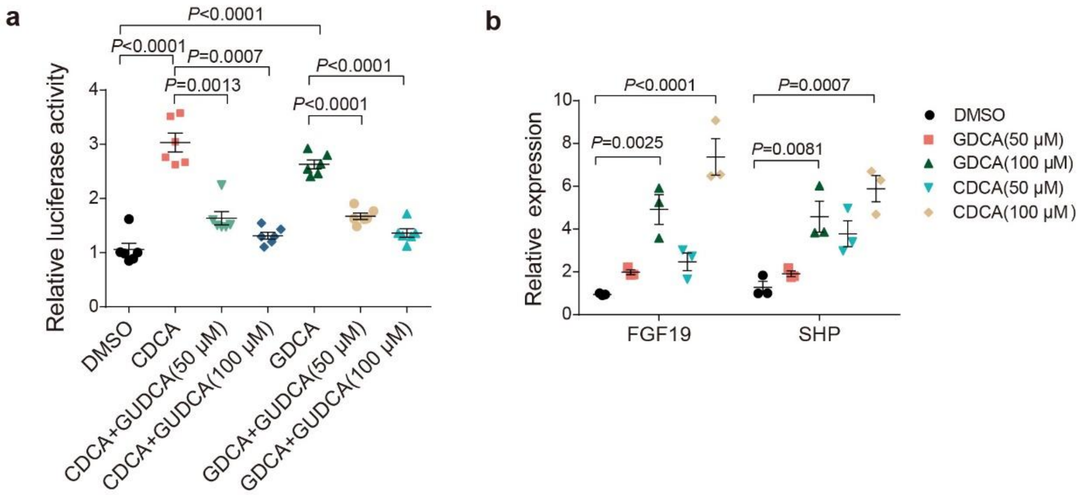

Supplementary Fig.11 GDCA acts as an agonist of FXR to induce the downstream gene expression of FXR. a Luciferase activity of GDCA or CDCA in the presence of different concentrations of GUDCA. n=6 per group. *P* values were determined by Welch ANOVA with Games-Howell's multiple comparisons test. **b** Relative mRNA expression of SHP and FGF19 in Caco-2 cells treated with different concentrations of GDCA or CDCA. n=3 per group. *P* values were determined by ANOVA with Tukey's multiple comparisons test. Data are presented as mean ± SEM for **a**, **b**. Source data are provided as a Source Data file.

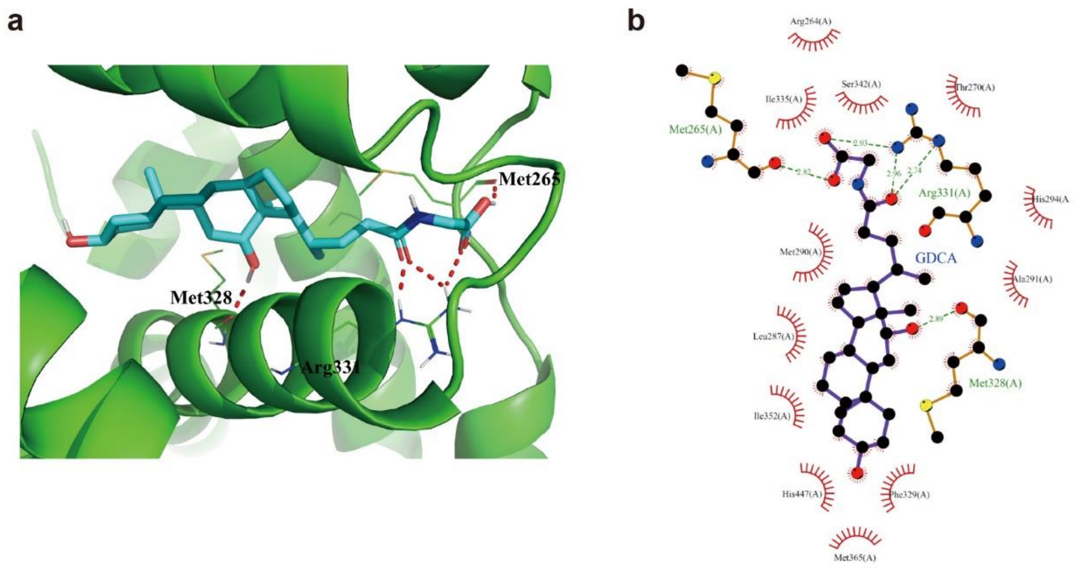

Supplementary Fig.12 Docking analysis. a The hydrogen binding interaction of GDCA with FXR. The hydrogen bonds are depicted as red dashed lines. **b** The 2D visualization and interactions between GDCA and FXR. The hydrogen bonds and the hydrophobic interactions were shown in green-dashed lines and red arcs, respectively.

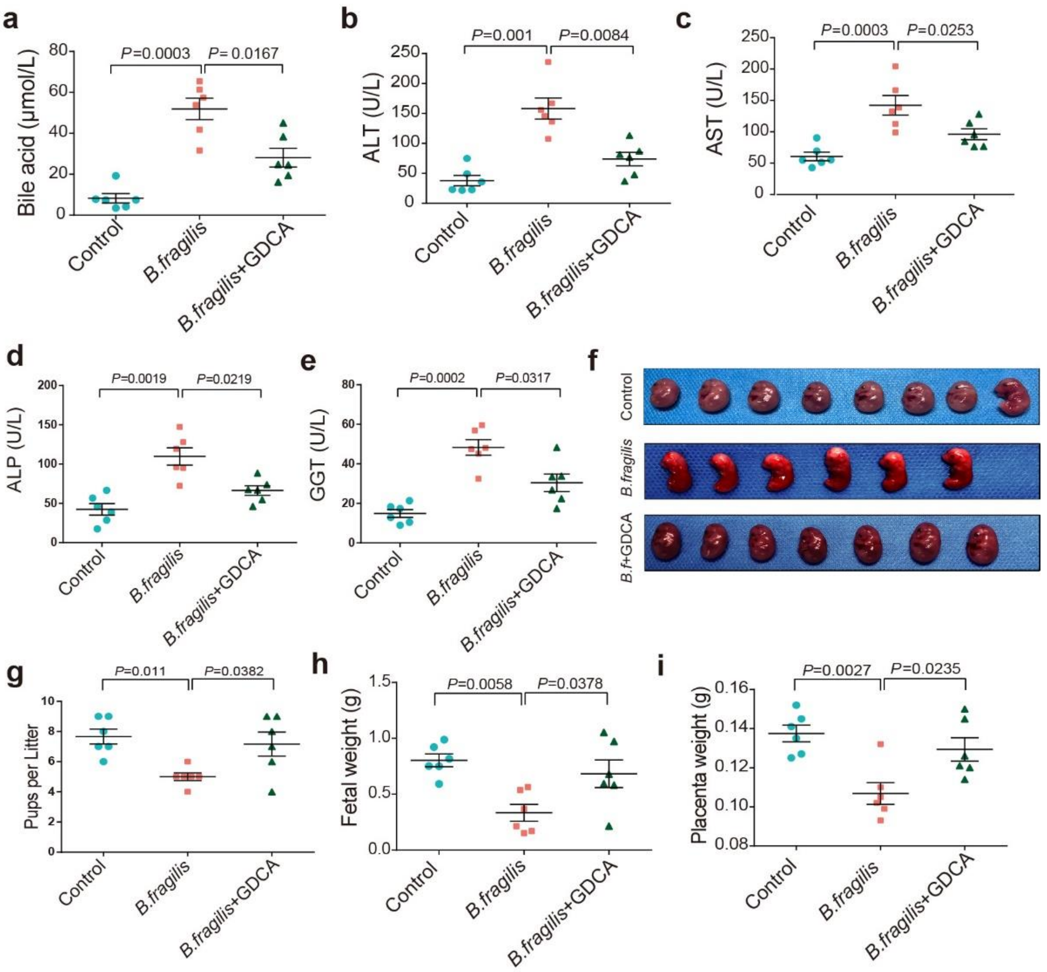

Supplementary Fig.13 Administration of GDCA improves *B. fragilis*-induced intrahepatic cholestasis in the pregnant mice model. a-e Analysis of serum levels of total bile acids, ALT, AST, ALP and GGT in each group (n=6 per group). *P* values were determined by Welch ANOVA with Games-Howell's multiple comparisons test. **f, g** Representative images of fetuses (**f**) and number of pups per litter (**g**) in each group. (n=6 per group). *P* value was determined by one-way ANOVA with Tukey's correction. **h, i** Fetal weight (**h**) and placenta weight (**i**) in each group (n=6 per group). *P* values were determined by one-way ANOVA with Tukey's multiple comparisons test. Data are presented as mean ± SEM for **a-e**, **g-i**. Source data are provided as a Source Data file.

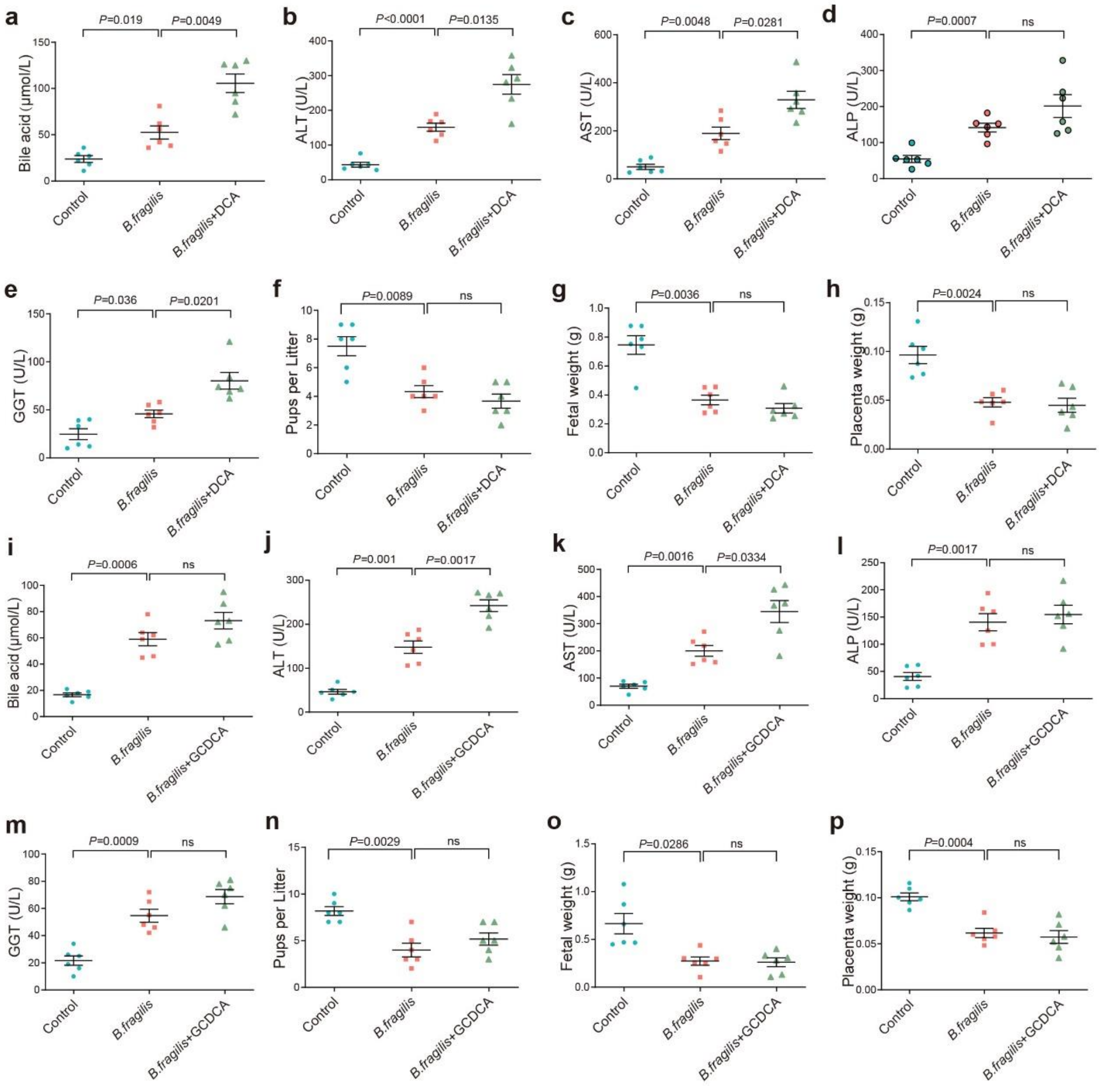

Supplementary Fig.14 Administration of DCA and GCDCA failed to mitigate *B. fragilis*-induced intrahepatic cholestasis. a-e Analysis of serum levels of total bile acids, ALT, AST, ALP and GGT in *B. fragilis* colonization mice with or without DCA administration (n=6). *P* values were determined by Welch ANOVA with Games-Howell's multiple comparisons test. **f-h** Number of pups per litter (**f**), fetal weight (**g**) and placenta weight (**h**) in *B. fragilis* colonization mice with or without DCA administration (n=6). *P* value was determined by Welch ANOVA with Games-Howell's multiple comparisons test. **i-m** Analysis of serum levels of total bile acids, ALT, AST, ALP and GGT in *B. fragilis* colonization mice with or without GCDCA administration

(n=6). *P* values were determined by Welch ANOVA with Games-Howell's multiple comparisons test. **n-p** Number of pups per litter (**n**), fetal weight (**o**) and placenta weight (**p**) in *B. fragilis* colonization mice with or without DCA administration (n=6). *P* value was determined by Welch ANOVA with Games-Howell's multiple comparisons test. Data are presented as mean ± SEM for **a-p**. Source data are provided as a Source Data file.

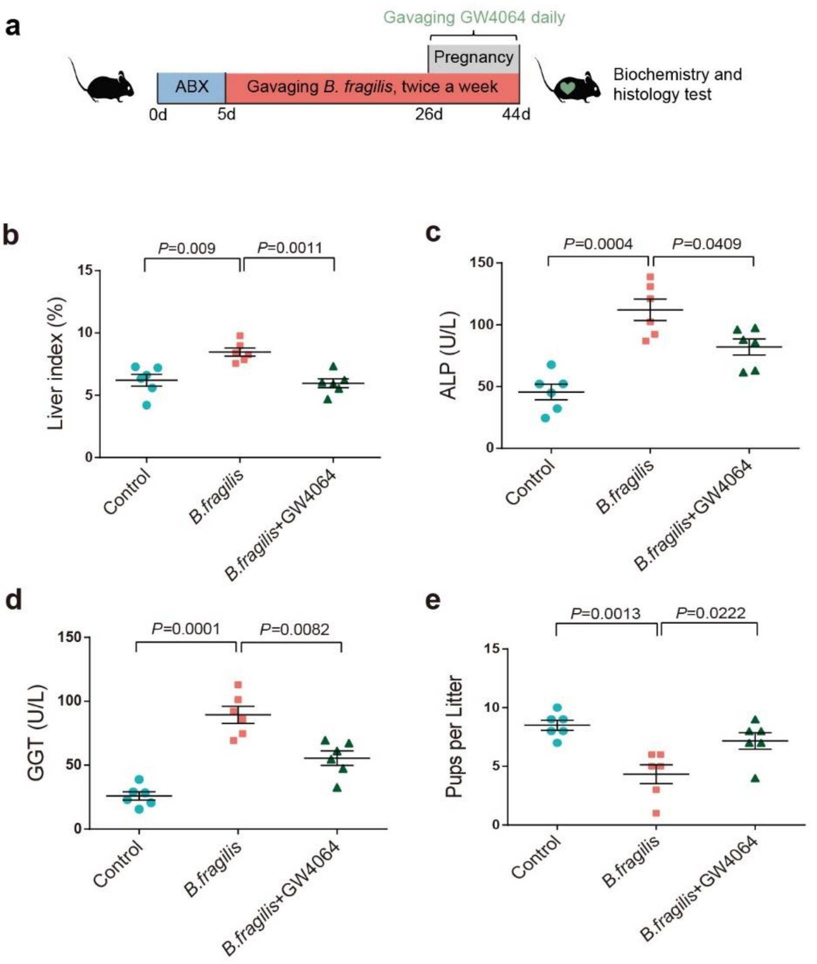

Supplementary Fig.15 FXR agonist GW4064 alleviates *B. fragilis*-induced cholestatic liver injury of pregnancy. a Experimental schematic: following antibiotics treatment, female recipient mice were gavaged with *B. fragilis* at a dose of 2×10^8 colony-forming units (cfu) per 200 μl sterile PBS twice a week until end of the pregnancy. Then, GW4064 (10 mg/kg/d) was gavaged at the beginning of pregnancy. Various tissues and samples were collected at E18d. **b-e** Liver index (**b**), serum levels of ALP (**c**) and GGT (**d**), and number of pups per litter (**e**) in each group. (n=6 per group). *P* values were determined by Welch ANOVA with Games-Howell's multiple comparisons test or one-way ANOVA with Tukey's multiple comparisons test. Data are presented as mean ± SEM for **b-e**. Source data are provided as a Source Data file.

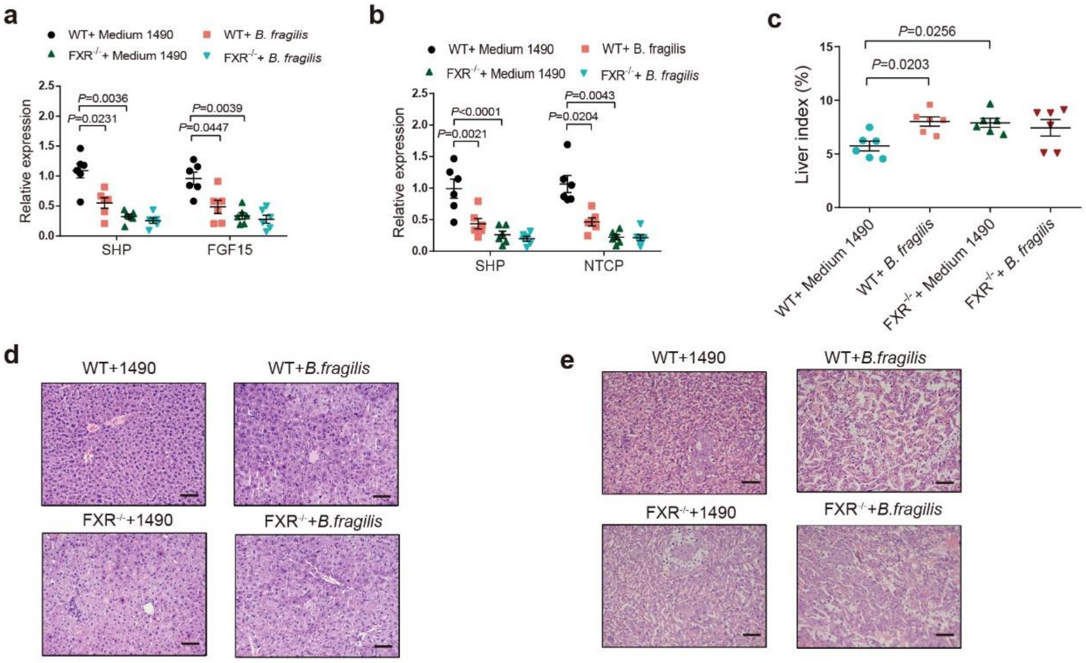

Supplementary Fig.16 FXR is essential for *B. fragilis*-induced intrahepatic cholestasis in pregnant mice model. WT and $FXR^{-/-}$ mice were colonized with *B. fragilis* or not. **a, b** Relative expression levels of the intestinal FXR target genes (**a**) and hepatic FXR target genes (**b**) in each group (n=6 per group). **c** Liver index in each group (n=6 per group). Data are presented as mean ± SEM for **a-c**. *P* values were determined by Welch ANOVA with Games-Howell's multiple comparisons test for **a-c**. **d, e** Representative images of H&E staining of livers and placenta. Scale bar: 50 μm. Source data are provided as a Source Data file.

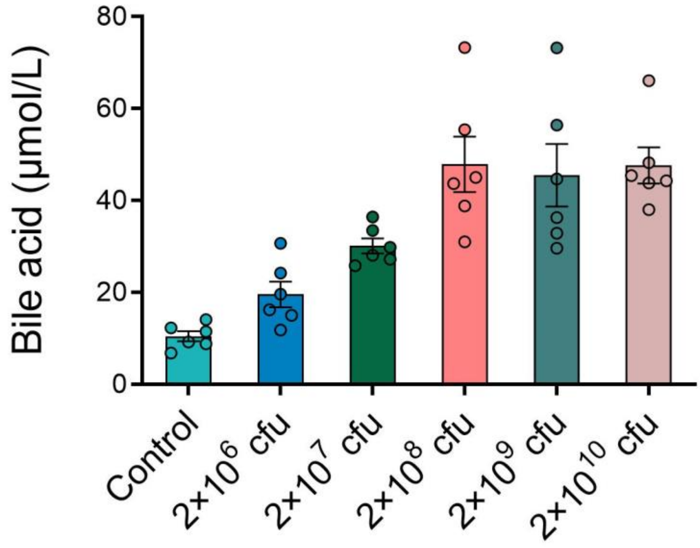

Supplementary Fig.17 Different doses of *B. fragilis* were used for colonization in pregnant mice. Serum levels of total bile acids in mice of each group were determined (n=6 per group). Data are presented as mean ± SEM. Source data are provided as a Source Data file.

Supplementary Tables

Supplementary Table 1. Baseline characteristics of the enrolled subjects.

	n=91	Health (n=41)	ICP(n=50)	*P* value
TBA	15.0 [7.90;20.5]	5.15 [4.70;5.70]	33.0 [23.7;40.8]	<0.001
age	29.0 [27.0;30.0]	29.0 [27.0;31.0]	28.0 [27.0;31.0]	0.885
gestational week	36.1 [35.1;37.1]	39.0 [38.0;39.2]	34.2 [33.4;35.4]	<0.001
height	160 [160;161]	160 [158;161]	160 [159;161]	0.636
weight	56.0 [52.5;58.0]	58.0 [53.0;61.0]	54.5 [52.0;58.0]	0.068
weight gain	11.0 [10.0;12.0]	11.0 [10.0;12.5]	10.8 [10.0;12.0]	0.974
BMI	21.4 [20.6;22.3]	22.3 [21.6;23.2]	20.7 [20.0;21.4]	0.030
TBIL	11.2 [10.1;12.5]	10.1 [7.40;11.2]	12.9 [10.8;17.1]	0.002
ALT	13.5 [12.0;27.6]	11.0 [10.0;12.0]	79.0 [35.0;127]	<0.001
AST	21.0 [18.0;26.0]	17.0 [17.0;20.0]	76.6 [28.0;118]	<0.001
ALP	76.3 [66.5;87.2]	77.8 [63.1;87.4]	75.8 [63.2;102]	0.380
GGT	41.0 [33.1;46.4]	28.3 [22.1;35.5]	62.0 [42.1;85.7]	<0.001
neoweight	3.00 [2.70;3.20]	3.20 [2.95;3.50]	2.64 [2.35;3.00]	<0.001
Apgar score	9.00 [9.00;9.00]	9.00 [9.00;9.00]	8.50 [8.00;9.00]	<0.001

Data are expressed in number (percentage, %) and median (IQR, interquartile range). *P* value was determined by two-tailed Student's t-test or two-tailed Mann-Whitney test.

Supplementary Table 2. Baseline characteristics of severe and mild ICP patients.

	Severe (n=20)	Mild (n=30)	*P* value
TBA	51.7 [45.7;57.1]	21.0 [17.3;28.7]	<0.001
age	27.0 [26.0;31.0]	30.0 [26.0;31.0]	0.584
gestational week	33.0 [32.1;35.4]	34.3 [33.5;35.6]	0.168
height	160 [159;164]	160 [158;161]	0.228
weight	56.0 [52.0;58.0]	52.0 [50.0;58.0]	0.306
weight gain	10.0 [8.00;12.0]	11.5 [10.0;13.0]	0.186
BMI	20.8 [19.6;22.7]	20.7 [20.0;21.4]	0.797
TBIL	11.8 [8.50;18.5]	14.6 [10.4;17.2]	0.342
ALT	114 [80.0;252]	40.0 [13.5;94.0]	0.020
AST	98.5 [50.0;142]	64.8 [18.0;102]	0.127
ALP	109 [66.5;206]	67.6 [56.3;84.5]	0.024
GGT	90.6 [61.0;125]	43.5 [35.0;75.2]	0.014
TC	6.50 [5.94;7.20]	6.38 [5.82;7.20]	0.874
TG	3.50 [2.91;4.74]	3.45 [2.94;4.20]	0.677
HDL	2.15 [1.92;2.48]	2.16 [1.98;2.20]	0.634
LDL	2.86 [2.31;3.54]	2.70 [2.36;3.42]	0.905
neoweight	2.29 [2.10;2.60]	3.05 [2.65;3.27]	0.002
Apgar score	8.00 [7.00;8.00]	9.00 [8.00;9.00]	0.003

Data are expressed in number (percentage, %) and median (IQR, interquartile range). *P* value was determined by two-tailed Student's t-test or two-tailed Mann-Whitney test.

Supplementary Table 3. Characteristics of FMT donors.

Donor ID	Age	BMI	TBA	TBIL	ALT	AST	ALP	GGT	neoweight	Apgar score
ICP1	31	20.3	55.8	62.3	157.5	118.3	205.6	230.3	2.7	6
ICP2	27	23.7	65.9	11.4	427.4	142.2	297.5	286.3	1.9	7
ICP3	31	18.9	37.1	6.9	45.1	33.5	75.1	58.4	2.3	9
ICP4	34	22.6	28.7	47.1	182.2	127.3	135.4	87.5	1.9	8
ICP5	26	19.3	34.2	12.2	296.8	245.1	149.6	45.2	2.7	7
ICP6	26	22.6	56.7	25.9	37.6	42.8	75.2	38.1	2.1	7
Control1	23	22.4	6.4	5.9	12.6	21.1	58.2	36.3	4.4	10
Control2	25	19.5	4.8	11.2	11.3	14.2	87.6	11.4	2.3	9
Control3	26	23.2	7.7	10.6	18.5	19.4	117.8	15.5	3.8	9
Control4	29	21.9	4.7	7.6	21.1	20.5	65.5	35.5	3.7	10
Control5	21	22.2	5.5	6.9	32.6	16.1	94.1	12.7	3.5	10
Control6	30	24.4	8.3	7.3	19.2	26.5	87.4	13.1	3.7	9

Supplementary Table 4. Primer sequences for Real-time PCR on mice.

Target genes		Sequences
Cyp7a1	Forward	5'- GTCCGGATATTCAAGGATGC-3'
	Reverse	5'- GGGAATGCCATTTACTTGGA -3'
Cyp27a1	Forward	5'- GCCTTGCACAAGGAAGTGACT -3'
	Reverse	5'- CGCAGGGTCTCCTTAATCACA -3'
Cyp8b1	Forward	5'- CCTCTGGACAAGGGTTTTGTG -3'
	Reverse	5'- GCACCGTGAAGACATCCCC -3'
Shp	Forward	5'- CGATCCTCTTCAACCCAGATG -3'
	Reverse	5'- AGGGCTCCAAGACTTCACACA -3'
Fxr	Forward	5'- TCCACAACCAAGTTTTGCAG -3'
	Reverse	5'- TCTCTGTTTGTTGTACGAATCCA-3'
Fgf15	Forward	5'- ACGTCCTTGATGGCAATCG -3'
	Reverse	5'- GAGGACCAAAACGAACGAAATT -3'
Bsep	Forward	5'- CTGCCAAGGATGCTAATGCA -3'
	Reverse	5'- CGATGGCTACCCTTTGCTTCT-3'
Ntcp	Forward	5'- ATGACCACCTGCTCCAGCTT -3'
	Reverse	5'- GCCTTTGTAGGGCACCTTGT -3'
Mrp2	Forward	5'- GCTTCCCATGGTGATCTCTT -3'
	Reverse	5'- ATCATCGCTTCCCAGGTACT -3'
TNF-α	Forward	5'-CCCACACCGTCAGCCGATTT-3'
	Reverse	5'-GTCTAAGTACTTGGGCAGATTGACC-3'
MCP-1	Forward	5'-TCAGCCAGATGCAGTTAACGC-3'
	Reverse	5'-TGATCCTCTTGTAGCTCTCCAGC-3'
IL-6	Forward	5'-GCCTTCTTGGGACTGATGCT-3'
	Reverse	5'-CTGCAAGTGCATCATCGTTGT-3'
18S	Forward	5'- CGATCCGAGGGCCTCACTA -3'
	Reverse	5'-AGTCCCTGCCCTTTGTACACA-3'

Supplementary Table 5. Primer sequences for Real-time PCR in human.

Target genes		Sequences
Shp	Forward	5'-TCAAGTCCATTCCGACCAGC -3'
	Reverse	5'- AAGAAGGCCAGCGATGTCAA-3'
Fgf19	Forward	5'-AGATCAAGGCAGTCGCTCTG -3'
	Reverse	5'- CGGATCTCCTCCTCGAAAGC-3'
18S	Forward	5'- GATATGCTCATGTGGTGTTG -3'
	Reverse	5'- AATCTTCTTCAGTCGCTCCA -3'

Original Gels and Blots for Supplementary figure 9a

Akt

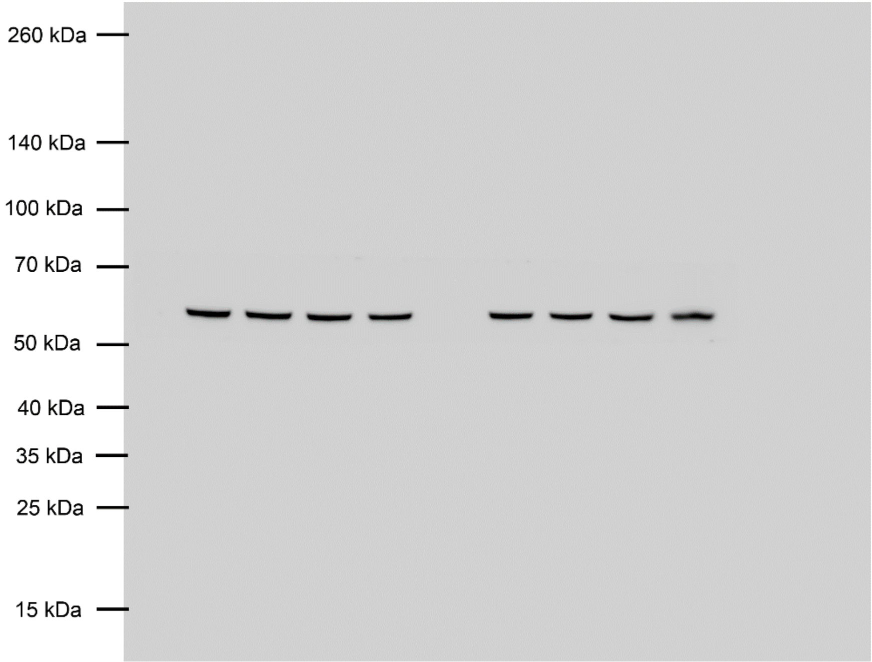

p-Akt

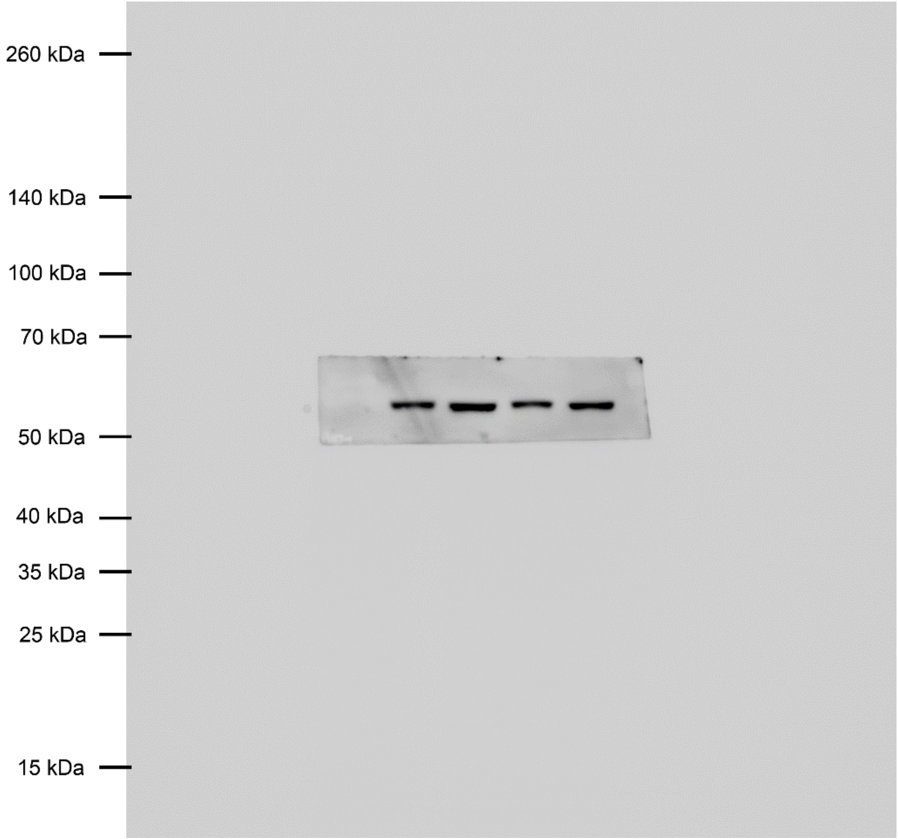

ERK

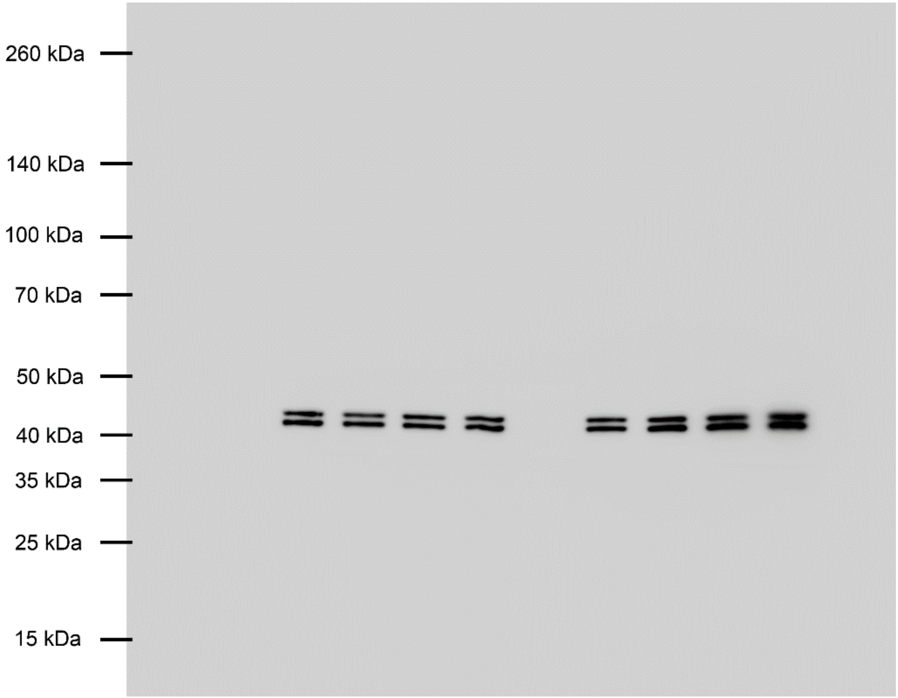
260 kDa
140 kDa
100 kDa
70 kDa
50 kDa
40 kDa
35 kDa
25 kDa
15 kDa

p-ERK

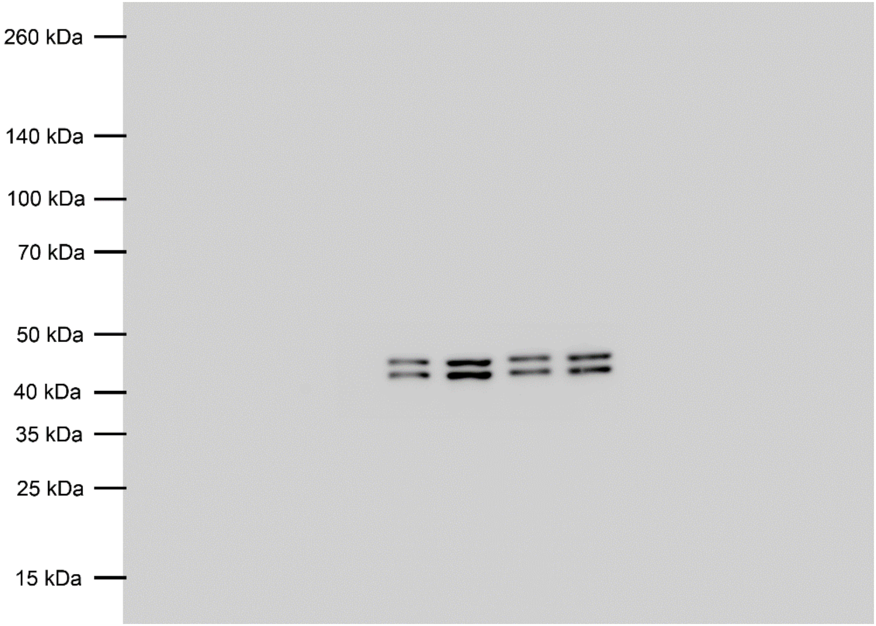
260 kDa
140 kDa
100 kDa
70 kDa
50 kDa
40 kDa
35 kDa
25 kDa
15 kDa
