# Supplementary material for: Gut microbiota alters host bile acid metabolism to contribute to intrahepatic cholestasis of pregnancy
Source: Nat Commun. 2023 Mar 9;14:1305. doi: 10.1038/s41467-023-36981-4 (PMC9998625; doi:10.1038/s41467-023-36981-4)
Supplement: Supplementary file 2 — Reporting Summary [file 41467_2023_36981_MOESM2_ESM.pdf]

nature portfolio

Corresponding author(s): Xia Chen, Shiming Yang

Last updated by author(s): Jan 24, 2023

Reporting Summary

Nature Portfolio wishes to improve the reproducibility of the work that we publish. This form provides structure for consistency and transparency in reporting. For further information on Nature Portfolio policies, see our Editorial Policies and the Editorial Policy Checklist.

Statistics

For all statistical analyses, confirm that the following items are present in the figure legend, table legend, main text, or Methods section.

n/a	Confirmed	
☐	☒	The exact sample size (*n*) for each experimental group/condition, given as a discrete number and unit of measurement
☐	☒	A statement on whether measurements were taken from distinct samples or whether the same sample was measured repeatedly
☐	☒	The statistical test(s) used AND whether they are one- or two-sided<br>*Only common tests should be described solely by name; describe more complex techniques in the Methods section.*
☒	☐	A description of all covariates tested
☐	☒	A description of any assumptions or corrections, such as tests of normality and adjustment for multiple comparisons
☐	☒	A full description of the statistical parameters including central tendency (e.g. means) or other basic estimates (e.g. regression coefficient) AND variation (e.g. standard deviation) or associated estimates of uncertainty (e.g. confidence intervals)
☐	☒	For null hypothesis testing, the test statistic (e.g. *F*, *t*, *r*) with confidence intervals, effect sizes, degrees of freedom and *P* value noted<br>*Give P values as exact values whenever suitable.*
☒	☐	For Bayesian analysis, information on the choice of priors and Markov chain Monte Carlo settings
☒	☐	For hierarchical and complex designs, identification of the appropriate level for tests and full reporting of outcomes
☒	☐	Estimates of effect sizes (e.g. Cohen's *d*, Pearson's *r*), indicating how they were calculated

Our web collection on statistics for biologists contains articles on many of the points above.

Software and code

Policy information about availability of computer code

Data collection	The characteristics of patients were recorded and calculated. The HE and immunohistochemistry results were collected by NIS-Elements 3.2. Multi Quant 2.1 software were used for bile acids data collection. 16S sequence data were obtained from an Illumina HiSeq platform (Illumina, San Diego, USA). Metagenomic data were obtained from an Illumina Novaseq 6000 platform. TR-FRET signal was measured in a Multi-Mode Microplate Reader. Luciferase activities were measured by Microplate Reader. Other data were collected with Microsoft Excel (2013) and GraphPad Prism software (v9).
Data analysis	The sample sizes were determined by power analysis using StatMate version 2.0 (GraphPad Software). GraphPad Prism version 9.0 (GraphPad Software) was used for statistical analysis. The Quantitative Insights into Microbial Ecology 2 (QIIME2, version 2019. 7) platform within a conda environment was used to process the 16S sequencing data. Metagenomic sequencing analysis process including trimming of low-quality bases, identification and masking of human DNA contamination, and removal of duplicated reads were performed by using kneaddata (version v0.6.1). Human DNA contamination was identified by aligning all raw reads to the human reference genome (hg19) using bowtie2 (version 2.3.5.1). Taxonomic annotation of metagenome and the abundance quantification were performed by MetaPhlAn (version 2.0).

For manuscripts utilizing custom algorithms or software that are central to the research but not yet described in published literature, software must be made available to editors and reviewers. We strongly encourage code deposition in a community repository (e.g. GitHub). See the Nature Portfolio guidelines for submitting code & software for further information.

Data

Policy information about availability of data

All manuscripts must include a data availability statement. This statement should provide the following information, where applicable:

- Accession codes, unique identifiers, or web links for publicly available datasets
- A description of any restrictions on data availability
- For clinical datasets or third party data, please ensure that the statement adheres to our policy

The 16S rRNA and metagenomic sequencing data generated in this study have been deposited in the European Nucleotide Archive database (https://www.ebi.ac.uk/ena) under accession code PRJEB42414 [https://www.ebi.ac.uk/ena/browser/view/PRJEB42414]. The raw clinical data of participants are protected and are not available due to data privacy laws. The data supporting the findings generated in this study are provided in the Supplementary Information and Source Data file. Source data are provided with this paper.

Human research participants

Policy information about studies involving human research participants and Sex and Gender in Research.

Reporting on sex and gender	Total 91 participants including 41 healthy pregnant women and 50 women with intrahepatic cholestasis of pregnancy were included in the study.
Population characteristics	50 individuals with ICP and 41 age, BMI and offspring gender matched healthy pregnant women were recruited from Chongqing and Guangdong province of China. There were 30 mild (TBA range 10-39.9 μmol/L) and 20 severe (TBA ≥40 μmol/L) ICP patients included.
Recruitment	ICP was diagnosed according to the Guidelines for diagnosis and treatment of intrahepatic cholestasis of pregnancy from China with the following criteria: unexplainable pruritus; elevated serum bile acids (≥10 μmol/L); no identifiable cause for liver dysfunction; resolution of symptoms and laboratory values postpartum. Exclusion criteria were as follows: preeclampsia, low platelets (HELLP) syndrome, acute fatty liver of pregnancy, active viral hepatitis and primary biliary cirrhosis; patients receiving any antibiotic or probiotics treatment within 1 months; patients with other pregnant complications such as pregnancy diabetes and hypertensive disorders. All pregnant women with ICP were first-visit patients and did not receive any treatment. 50 individuals with ICP and 41 age, BMI and offspring gender matched healthy pregnant women were recruited from Chongqing and Guangdong province of China.
Ethics oversight	The study was approved by the Ethics Committee of Xinqiao Hospital, Army Medical University (Approved No. 2020-146-01). Written informed consents for participating this study and publishing individual information were obtained from all participants. Participants didn't receive cash remuneration.

Note that full information on the approval of the study protocol must also be provided in the manuscript.

Field-specific reporting

Please select the one below that is the best fit for your research. If you are not sure, read the appropriate sections before making your selection.

☒ Life sciences ☐ Behavioural & social sciences ☐ Ecological, evolutionary & environmental sciences

For a reference copy of the document with all sections, see nature.com/documents/nr-reporting-summary-flat.pdf

Life sciences study design

All studies must disclose on these points even when the disclosure is negative.

Sample size	Sample sized were determined based on pilot studies, or based on prior experience with similar studies. Animals' number calculation was by "resource equation" method. According to this method, a value "E" is measured. The value of E should lie between 10 and 20. If E is less than 10 then adding more animals will increase the chance of getting more significant result, but if it is more than 20 then adding more animals will not increase the chance of getting significant results. It is considerable to be applicable to all animal experiments. Any sample size, which keeps E between 10 and 20 should be considered as an adequate. E can be measured by following formula: E = Total number of animals – Total number of groups).
Data exclusions	No data were excluded.
Replication	All experimental findings were pooled from three independent experiments. All replication were successful.
Randomization	Human participants were randomly selected under the criteria in the Methods. Six- to eight-week-old female mice were randomly divided into indicated experimental groups, with at least 6 mice per group, and the mice did not show differences before treatment.

Blinding	For HE or IHC staining and analysis, pathologists were blinded to group allocation. The investigators were not blinded to other data collection or data analysis because of no subjective assessments were included.

Reporting for specific materials, systems and methods

We require information from authors about some types of materials, experimental systems and methods used in many studies. Here, indicate whether each material, system or method listed is relevant to your study. If you are not sure if a list item applies to your research, read the appropriate section before selecting a response.

Materials & experimental systems

n/a	Involved in the study
☐	☒ Antibodies
☐	☒ Eukaryotic cell lines
☒	☐ Palaeontology and archaeology
☐	☒ Animals and other organisms
☒	☐ Clinical data
☒	☐ Dual use research of concern

Methods

n/a	Involved in the study
☒	☐ ChIP-seq
☒	☐ Flow cytometry
☒	☐ MRI-based neuroimaging

Antibodies

Antibodies used	Western blot was performed using the primary antibodies for p-ERK (Cat#4370, Cell Signaling, 1:1000), ERK (Cat#4695, Cell Signaling, 1:1000), p-AKT (Cat#4060, Cell Signaling, 1:1000), and AKT (Cat#9272, Cell Signaling, 1:1000), and the secondary antibody conjugated with horseradish peroxidase (Cat# A0208, Beyotime, 1: 2000). Immunohistochemistry was performed using the primary antibodies for Cyp7a1 (Cat#bs-21430R, Bioss, 1:100), Cyp8b1 (Cat#bs-14165R, Bioss, 1:100), Cyp27a1 (Cat#bs-5049R, Bioss, 1:100), MRP2 (Cat#bs-1092R, Bioss, 1:100) and BSEP (Cat#bs-12440R, Bioss, 1:100).
Validation	The antibodies used in our study were commercially purchased and have been validated by the manufacturers and used according to the manufacturers' instructions. Validation data are available from the respective vendor's websites.

Eukaryotic cell lines

Policy information about cell lines and Sex and Gender in Research

Cell line source(s)	Caco-2 (Cat# HTB-37, ATCC) and HEK293 cells (Cat#CRL-3216, ATCC) were purchased from ATCC.
Authentication	The cell lines were authenticated by the manufacturer.
Mycoplasma contamination	The cell lines were tested and negative for mycoplasma contamination.
Commonly misidentified lines (See ICLAC register)	No commonly misidentified cell lines were used in this study.

Animals and other research organisms

Policy information about studies involving animals; ARRIVE guidelines recommended for reporting animal research, and Sex and Gender in Research

Laboratory animals	B6.129X1(FVB)-Nr1h4tm1Gonz/J mice were obtained from Jackson Laboratory (Cat# 00724). C57BL/6 mice were from Vital River Laboratories (Beijing, China). All animals involved in our experiments were female mice aged 6-8 weeks and weighed 20-22g.
Wild animals	There are no wild animals involved in our study.
Reporting on sex	Female mice were considered in this study and reported in the Methods.
Field-collected samples	There are no field-collected samples involved in our study.
Ethics oversight	All animal protocols were approved by the Animal Care and Use Committee of the Army Medical University and adhered to the Animal Ethics Statement (Approved No. AMUWEC2020197)

Note that full information on the approval of the study protocol must also be provided in the manuscript.
